# Genotype-derived interpretive categories of drug response in 908 pharmacogenomic reports from Chinese patients with type 2 diabetes: A retrospective single-centre study

**DOI:** 10.1371/journal.pone.0351903

**Published:** 2026-11-05

**Authors:** Ye Liu, Chun-xiao Yu, Li Lu

**Affiliations:** 1 Department of Geriatrics, Shandong Zaozhuang Municipal Hospital, Zaozhuang, Shandong, China; 2 Department of Endocrinology, Shandong Health Group Zaozhuang Central Hospital, Zaozhuang, Shandong, China; 3 Department of Urology, Shandong Zaozhuang Municipal Hospital, Zaozhuang, Shandong, China; Karmanos Cancer Institute, Wayne State University School of Medicine, UNITED STATES OF AMERICA

## Abstract

Pharmacogenomic testing is increasingly ordered in routine diabetes care, but the reports it generates are heterogeneous and are not in a form that can be analysed systematically. We examined how genotype is translated into interpretive categories of drug response in a real-world Chinese cohort, and how consistently that translation is applied. We analysed 908 de-identified pharmacogenomic reports issued for patients with type 2 diabetes at a single centre between July 2022 and April 2024. An interpretation rulebook, taken from the standing appendix that the testing laboratory attached to every report and completed by reconstructing the rules that appendix does not state, mapped six single-nucleotide polymorphisms onto eight genotype-derived interpretive categories. These are classifications, not measured outcomes: no glycaemic response, hypoglycaemia, adverse event, treatment discontinuation or dose modification was recorded, so no category could be checked against outcome. Allele frequencies matched gnomAD East Asian data except at the risk loci, where the risk alleles were enriched, as expected in a case-only cohort. Each pre-defined variant was the statistical driver of its corresponding category, with odds ratios inflated by quasi-complete separation because the coding rules are deterministic. Across 5,983 category assignments the rulebook reproduced 5,940 (99.3%), leaving 43 discordant assignments in 28 reports; a structured audit makes most of them consistent with a mismatch between the genotype printed on a report and the genotype its interpretation was generated from. The sulfonylurea efficacy category was assigned by two different rules over the study period, and the “increased efficacy” level appeared only in reports from the later, six-locus panel, where it tracked reduced-function *CYP2C9* genotypes. Nothing in the report indicated which rule had been applied. Interpretation drifts as assays are revised, and writing the rulebook down and versioning it is what makes that drift visible.

## Introduction

Type 2 diabetes mellitus (T2DM) is a significant and growing global public health challenge, with an estimated 537 million people affected worldwide in 2021. China, in particular, has the largest number of individuals with diabetes, underscoring the urgent need for effective prevention and management strategies. Management is centred on glycaemic control through lifestyle interventions and pharmacological therapy [1]. Oral antidiabetic drugs are a cornerstone of this treatment paradigm. Clinical practice is nonetheless marked by wide inter-individual variability in drug efficacy and adverse reactions, which motivates the pursuit of pharmacological precision medicine in diabetes [2,3]. Pharmacogenomics, which studies the influence of genetic variation on drug response, offers a promising pathway to tailor drug selection and dosage to a patient’s unique genetic profile, thereby advancing the goal of precision medicine for T2DM.

A substantial body of evidence has established associations between specific genetic polymorphisms and both T2DM susceptibility and drug response. For disease susceptibility, robust genome-wide association studies have implicated variants in *TCF7L2*, *IRS1*, and *CDKAL1* among the most reproducible common risk loci [4,5]. For pharmacogenetics, *CYP2C9* polymorphisms alter sulfonylurea metabolism and are associated with variability in glycaemic response and hypoglycaemia risk [6]. Transporter genetics also play a critical role: *SLCO1B1* variants markedly influence repaglinide pharmacokinetics, an effect replicated in Han Chinese cohorts, while common variants near *ATM* have been linked to metformin glycaemic response in large discovery and replication studies [7,8]. Notably, meta-analytic work indicates that the impact of pharmacokinetic transporter variants on metformin glycaemic response can be modest and context-dependent, underscoring the need for carefully standardized phenotyping [9]. Beyond these loci, *TCF7L2* risk alleles have been associated with reduced sulfonylurea effectiveness across multiple studies, consistent with impaired β-cell function and incretin signalling [10].

Despite this wealth of knowledge, the translation of findings from heterogeneous real-world genetic reports into standardized, analysable variables remains a critical implementation bottleneck. While authoritative resources such as PharmGKB and the Clinical Pharmacogenetics Implementation Consortium (CPIC) provide the essential “evidence base” and high-level clinical recommendations, there remains a distinct “operational gap” in how these guidelines are systematically applied to raw datasets [11]. Current guidelines effectively define what to do when a metabolizer status is known (e.g., dose reduction for a Poor Metabolizer), but they often lack the deterministic logic required to batch-process raw genotype data into discrete, uniformly coded interpretive categories. That logic must cope with multiple loci and with varying data quality.

Consequently, our proposed framework functions as a translational “middleware.” Unlike high-level evidence repositories, our “Interpretation Rulebook” provides the specific, standardized logic layer necessary to convert raw genetic inputs into uniformly coded interpretive categories. This approach mimics the architecture required for Electronic Health Record (EHR)-integrated Clinical Decision Support Systems (CDSS), moving beyond static guidelines to a standardized data model capable of handling the complexities of real-world Chinese cohort data. Whether the categories such a model produces carry clinical information is a separate question, and one this study does not answer. Furthermore, ancestry-related transferability issues in genetic prediction highlight the importance of generating evidence in underrepresented populations, including East Asians/Chinese [12], to ensure that global guidelines are locally applicable. In parallel, while polygenic risk scores (PRS) have shown promise for stratifying T2DM risk at scale, ensuring equitable performance across ancestries remains an active area of methodological development [13,14].

To address this gap between genetic knowledge and clinical application, this study was designed to systematically evaluate the pharmacogenomic landscape in a real-world cohort of 908 pharmacogenomic reports from 887 Chinese patients with type 2 diabetes mellitus. We first set out the standardized “Interpretation Rulebook” used to convert raw genotype data into eight genotype-derived interpretive categories, and the laboratory document from which it was transcribed. We then pursued three objectives. The first was to describe the frequency distribution of the key polymorphisms and to compare it with East Asian reference data. The second was to test how consistently the rulebook had in fact been applied across the cohort. The third was to construct a polygenic risk score and examine what it does and does not establish. Our aim is a documented and internally consistent framework for standardizing heterogeneous real-world genetic data in the Chinese population. We make no claim of clinical utility for the categories that framework produces: no independent external, prospective or outcome-based validation was undertaken.

## Materials and methods

### Study design and population

This retrospective cohort study analysed de-identified records from patients who underwent pharmacogenomic testing at the Endocrinology Department of Shandong Health Group Zaozhuang Central Hospital between July 2022 and April 2024. All patients with a clinical diagnosis of T2DM and at least one successfully genotyped locus were eligible. No age restriction was applied, so the cohort spans 9–93 years and includes five participants younger than 18 years. Age, sex and referring department were extracted from the laboratory information system linked to the testing service. Patients with incomplete demographic data or indeterminate genotype calls were excluded. Clinical definitions and treatment targets followed the Standards of Care in Diabetes 2025 [15]. The unit of analysis is the issued report record rather than the person; 20 patients contributed more than one record, as set out in the limitations.

Two versions of the genotyping panel were in use during the study period, and this determines the structure of the missing data. A four-locus panel covering *CYP2C9* rs1057910, *TCF7L2* rs7903146, *IRS1* rs1801278 and *CDKAL1* rs7754840 was used for 427 records. An expanded six-locus panel that additionally assayed *SLCO1B1* rs4149056 and *CDKAL1* rs7756992 was used for the remaining 481. In 4 of those 481 records an early configuration of the expanded panel assayed *FGG* rs2066865 in place of *CDKAL1* rs7756992, so six loci were assayed per patient throughout and seven distinct loci were assayed across the two panel versions, but never seven in one patient. *FGG* rs2066865 is not analysed further, because it was genotyped in only 4 records. The changeover was a clean calendar-time one. The laboratory line-level export carries an explicit annotation recording that the test assayed six loci from 29 April 2023 and four loci before that date, and among the 801 records that could be matched to that export every four-locus record was tested between 12 July 2022 and 28 April 2023 and every six-locus record between 29 April 2023 and 10 April 2024. There is no overlap. Missingness at *SLCO1B1* rs4149056 and *CDKAL1* rs7756992 is therefore structural rather than random, because it reflects which panel version issued a record, and panel version in turn is fixed by calendar time. Analyses involving those loci are restricted to the six-locus subgroup, and this restriction is stated wherever it applies.

### Ethics statement

The study protocol complied with the Declaration of Helsinki. Ethical approval was granted on 27 April 2022 by the Medical Ethics Committee of Zaozhuang Mining Group Central Hospital (approval number 2022-Ethics Review-02, the certified English rendering of the Chinese original 2022-伦理审查-02), at a full-board initial review of the project “Clinical application study on genetic risk assessment and medication guidance for type 2 diabetes mellitus,” with Li Lu, of the Department of Endocrinology, as principal investigator. Two institutions are involved and they are distinct: the approving body is the ethics committee of Zaozhuang Mining Group Central Hospital, whereas the pharmacogenomic testing was carried out at, and the records analysed here were generated by, the Department of Endocrinology of Shandong Health Group Zaozhuang Central Hospital. The documents the committee reviewed included the study protocol and a written application for waiver of informed consent, and the committee granted that waiver; the records analysed include five participants younger than 18 years, none of whom was contacted and from none of whom was an additional specimen collected. Two features of the approval bear stating explicitly. It carries a validity period of 27 April 2022–26 April 2023 with a twelve-month continuing-review cycle, whereas the records analysed here extend to April 2024; the genotyping was ordered and performed throughout as part of routine clinical care rather than as research recruitment, and what the approval covers, and what is reported here, is the retrospective analysis of the resulting de-identified records. And the analysis was not conducted on anonymous data from the outset: extracting the records and establishing which of them belonged to the same patient required access to identifiers, which was done by the study team under the approval. Names and laboratory accession numbers were then removed, the deposited dataset carries no name, accession number or date, and the key linking study codes to accession numbers is held by the study team and is not published.

### Genotyping, variant curation, and category assignment

Genotyping was performed as part of routine clinical care rather than for research purposes. Whole-blood samples were referred by the Endocrinology Department to an independent clinical laboratory (Jinan Fuyou Medical Laboratory, Jinan, Shandong, China), which performed the assays and issued the interpretive reports from which the categories analysed here were taken. The laboratory did so as part of the clinical service and had no role in the design of this analysis, in its interpretation, or in the decision to publish. Testing was carried out on a Tianlong TL988 real-time fluorescence PCR system (Xi’an Tianlong Science and Technology, Xi’an, China), the instrument named on those reports that recorded the testing equipment. The assay chemistry, the reagent manufacturer and the reagent lot were not documented on any report and could not be recovered retrospectively; this remains the principal reproducibility limitation of the source data, and it is not one we can repair. The loci were pre-specified from prior evidence and comprised disease-risk variants (*TCF7L2*, *IRS1*, *CDKAL1*) together with pharmacogenetic loci relevant to oral antidiabetic therapy (*CYP2C9*, *SLCO1B1*). The interpretive rules applied here were not developed for this study; they were taken from the laboratory’s own interpretation document, which is described below and set out row by row, with the evidence level attached to each mapping, in S1 Table. Where this paper refers to the wider evidence framework for these gene-drug pairs it draws on PharmGKB and on published guideline frameworks, including that of the Dutch Pharmacogenetics Working Group [11,16]. The genotype call rates, the handling of missing, indeterminate and repeat results, and the quantitative criteria under which a locus would have been excluded are set out in the next subsection.

The rulebook transformed raw genotypes into eight genotype-derived interpretive categories: T2DM risk, insulin resistance risk, sulfonylurea (SU) efficacy, SU adverse drug reaction (ADR) risk, dipeptidyl peptidase-4 inhibitor (DPP-4i) efficacy, metformin efficacy, repaglinide efficacy and rosiglitazone efficacy. The names are those printed on the issued reports; each names a classification, not an observed response. Reduced-function *CYP2C9* genotypes were mapped to increased SU-related ADR risk, *SLCO1B1* variation determined the repaglinide efficacy category, and *TCF7L2* risk alleles were treated as markers of reduced insulin secretory capacity [17].

### Genotyping quality control

Genotypes were called by the referring laboratory on the Tianlong TL988 system and transcribed onto the issued report as a single called genotype for each locus the panel assayed. The underlying amplification data were not part of the issued report and were not available to us, so no call could be re-examined or re-called; every genotype analysed here is the genotype as issued.

Call rates were computed over the records whose panel assayed each locus. Among the 908 analysed records, a genotype was returned at *CYP2C9* rs1057910, *TCF7L2* rs7903146 and *IRS1* rs1801278 for 908 of 908 records (100%), and at *CDKAL1* rs7754840 for 907 of 908 (99.9%). Within the six-locus panel, *SLCO1B1* rs4149056 returned a genotype for 481 of 481 records (100%) and *CDKAL1* rs7756992 for 477 of the 477 records whose panel assayed it (100%); the 4 remaining six-locus records assayed *FGG* rs2066865 in its place and returned a genotype in 4 of 4. Taken across every locus that a record’s panel assayed, 4,593 of 4,594 locus-level results returned a genotype, an overall call rate of 99.98%.

The single exception was one six-locus record with no *CDKAL1* rs7754840 result. That locus has no row for the record in the laboratory line-level export, so a locus that was never attempted cannot be distinguished retrospectively from a call that was attempted and failed. The record was handled by complete-case exclusion from every analysis that uses rs7754840. No genotype in any of the 908 reports was recorded as indeterminate, ambiguous or failed, so no such call had to be handled; had one occurred it would have been excluded in the same way, but we did not have to fix that rule in advance because the situation never arose. No genotype was imputed at any point.

Twenty patients contributed more than one analysed record, and these repeats are the only internal check on assay reproducibility available to us. Comparing the repeat records of each such patient locus by locus gave 94 pairwise comparisons, of which 91 agreed (96.8%). The three disagreements arose in two patients, and all three were at *CDKAL1* and all three lay between a heterozygote and a homozygote: rs7754840 GG against CG together with rs7756992 AA against AG in one patient, and rs7754840 GG against CG in the other. Applying the same comparison to the full laboratory export, in which 29 patients had repeat samples, gave 132 of 138 concordant comparisons (95.7%). Repeat records were analysed as issued and were not reconciled against one another.

The previous version of this manuscript stated the exclusion criteria only qualitatively; both are given here in full and were applied uniformly to every locus. A locus was excluded for low call rate if fewer than 95% of the records whose panel assayed it returned a genotype. A locus was excluded for extreme deviation from Hardy-Weinberg equilibrium (HWE) if its chi-squared P value fell below 0.05/6 = 0.0083, a Bonferroni threshold over the six analysed loci. No locus met either criterion, and no locus was excluded on quality-control grounds. *FGG* rs2066865 was excluded from the association analyses for a different reason: it was genotyped in only 4 records, too few for a frequency, equilibrium or association statistic to carry any weight. HWE was tested with the chi-squared test on 1 degree of freedom, over all 908 records and again over one record per patient, which leaves 887 records and per-locus denominators of 887, 887, 887, 886, 461 and 457; the frequencies and test results for every locus are given in S2 Table. S3 Table gives the call rates, the repeat-testing comparison and the exclusion criteria, locus by locus.

### Definition of the genotype-derived interpretive categories

The primary variables were the eight interpretive categories assigned from genotype by the pre-specified rulebook. They were defined as binary or ordered categorical variables covering the genotype-derived risk of T2DM and of insulin resistance, the efficacy category for each of five oral agents, and SU-related ADR risk. We refer to them throughout as genotype-derived interpretive categories, and not as phenotypes, because nothing about a patient’s drug response was measured.

These categories are standardized rule-based classifications rather than measured clinical outcomes. No measured drug-response outcome was available for any patient in this cohort: glycaemic response, hypoglycaemia, adverse events, treatment discontinuation and dose modification were not recorded, so no category could be checked against outcome and none should be read as treatment guidance. The association analyses that follow therefore assess the internal consistency of the classification framework; they are not attempts to discover novel biological links.

The rulebook states, for each assayed locus, the interpretive category assigned to each genotype. The *TCF7L2* rs7903146 T allele, which has been linked to reduced insulin secretion, maps to an “Increased” T2DM risk category [18,19]. The *IRS1* rs1801278 minor allele maps to increased insulin resistance risk [20]. Reduced-function *CYP2C9* genotypes map to increased SU-ADR risk, and *CDKAL1* rs7754840 and rs7756992 inform both T2DM risk and the DPP-4i efficacy category [21,22]. *SLCO1B1* rs4149056 informs the repaglinide and rosiglitazone efficacy categories. Five of the eight categories are assigned from a single locus. Three are composites: T2DM risk, SU efficacy and DPP-4i efficacy each draw on more than one locus, and each therefore requires a precedence rule.

Categories were coded on a three-level ordinal scale. “Normal” means that the genotype fell in the reference class for that mapping. “Increased” or “Reduced” means that it fell in a non-reference class, in the direction the rule specifies. The levels are labels for a classification. They carry no dosing, monitoring or drug-selection recommendation, and for the reason given above none of them has been checked against a measured drug-response outcome. Table 1 gives the final genotype-to-category specification, and S1 Table gives the stated basis of each level together with the grading of the supporting evidence.

**Table 1:** The Interpretation Rulebook: genotype-to-category mapping, evidence level and panel version.

Gene	Locus (rsID)	Genotype	Interpretive category	Category level	Evidence level	Panel versions in which the rule was applied
*TCF7L2*	rs7903146	CC	T2DM risk	Normal	Not graded	Four- and six-locus
		CT	T2DM risk	Increased	Not graded	Four- and six-locus
		TT	T2DM risk	Increased	Not graded	Four- and six-locus
		CC	SU efficacy	Normal	PharmGKB level 3	Four- and six-locus
		CT	SU efficacy	Reduced	PharmGKB level 3	Four- and six-locus
		TT	SU efficacy	Reduced	PharmGKB level 3	Four- and six-locus
*IRS1*	rs1801278	CC	T2DM risk	Normal	Not graded	Four- and six-locus
		CT	T2DM risk	Increased	Not graded	Four- and six-locus
		TT	T2DM risk	Increased	Not graded	Four- and six-locus
		CC	Insulin resistance risk	Normal	Not graded	Four- and six-locus
		CT	Insulin resistance risk	Increased	Not graded	Four- and six-locus
		TT	Insulin resistance risk	Increased	Not graded	Four- and six-locus
		CC	SU efficacy	Normal	PharmGKB level 3	Six-locus only
		CT	SU efficacy	Reduced	PharmGKB level 3	Six-locus only
		TT	SU efficacy	Reduced	PharmGKB level 3	Six-locus only
		CC	Metformin efficacy	Normal	PharmGKB level 3	Six-locus only
		CT	Metformin efficacy	Reduced	PharmGKB level 3	Six-locus only
		TT	Metformin efficacy	Reduced	PharmGKB level 3	Six-locus only
*CDKAL1*	rs7756992	AA	T2DM risk	Normal	Not graded	Six-locus only
		AG	T2DM risk	Increased	Not graded	Six-locus only
		GG	T2DM risk	Increased	Not graded	Six-locus only
		AA	DPP-4i efficacy	Reduced	PharmGKB level 3	Six-locus only
		AG	DPP-4i efficacy	Normal	PharmGKB level 3	Six-locus only
		GG	DPP-4i efficacy	Increased	PharmGKB level 3	Six-locus only
*CDKAL1*	rs7754840	GG	T2DM risk	Normal	Not graded	Four- and six-locus
		CG	T2DM risk	Increased	Not graded	Four- and six-locus
		CC	T2DM risk	Increased	Not graded	Four- and six-locus
		GG	DPP-4i efficacy	Reduced	PharmGKB level 3	Four- and six-locus
		CG	DPP-4i efficacy	Normal	PharmGKB level 3	Four- and six-locus
		CC	DPP-4i efficacy	Increased	PharmGKB level 3	Four- and six-locus
*CYP2C9*	rs1057910	*1/*1 (AA)	SU ADR risk	Normal	PharmGKB level 3	Four- and six-locus
		*1/*3 (AC)	SU ADR risk	Increased	PharmGKB level 3	Four- and six-locus
		*3/*3 (CC)	SU ADR risk	Increased	PharmGKB level 3	Four- and six-locus
		*1/*1 (AA)	SU efficacy	Normal	PharmGKB level 3	Six-locus only; reconstructed
		*1/*3 (AC)	SU efficacy	Increased	PharmGKB level 3	Six-locus only; reconstructed
		*3/*3 (CC)	SU efficacy	Increased	PharmGKB level 3	Six-locus only; reconstructed
*SLCO1B1*	rs4149056	TT	Repaglinide efficacy	Normal	PharmGKB level 3	Six-locus only
		CT	Repaglinide efficacy	Increased	PharmGKB level 3	Six-locus only
		CC	Repaglinide efficacy	Increased	PharmGKB level 3	Six-locus only
		TT	Rosiglitazone efficacy	Normal	PharmGKB level 3	Six-locus only
		CT	Rosiglitazone efficacy	Increased	PharmGKB level 3	Six-locus only
		CC	Rosiglitazone efficacy	Increased	PharmGKB level 3	Six-locus only

SU, sulfonylurea; ADR, adverse drug reaction; DPP-4i, dipeptidyl peptidase-4 inhibitor; T2DM, type 2 diabetes mellitus. The table was taken from the standing appendix carried by every issued report. Evidence level is the grade printed in that appendix, except for the three rows marked “reconstructed”, which carry the grade the appendix attaches to *CYP2C9* for the sulfonylureas. The PharmGKB clinical annotation scale runs 1A, 1B, 2A, 2B, 3 and 4 in decreasing order of evidential strength, and level 3 denotes a low level of evidence: a single study, an unreplicated association, case reports, non-significant studies, or in vitro or functional evidence. Mappings marked “Not graded” carry no PharmGKB level and rest on locus-specific literature. Every value in the table is an interpretive category assigned by rule from genotype; none is a measured drug response, and no category level implies a dosing, monitoring or drug-selection recommendation. Four precedence rules resolve the three composite categories. (i) Composite T2DM risk takes the highest risk level across the contributing loci, so the category is Increased if any contributing locus carries a risk genotype. (ii) Composite DPP-4i efficacy is determined by *CDKAL1* rs7754840; where rs7754840 is the CG heterozygote, which maps to Normal, *CDKAL1* rs7756992 determines the level. (iii) For SU efficacy a reduced-function *TCF7L2* or *IRS1* genotype takes precedence over the increase assigned by a reduced-function *CYP2C9* genotype. (iv) Where no rule applied, the category was left missing rather than imputed. These four precedence rules are a reconstruction: the appendix states the interpretation of each genotype at each locus separately and does not state how contributing loci are combined. The last column records the panel versions in which each rule was applied to issued reports. For metformin, repaglinide and rosiglitazone efficacy a six-locus entry means that the category was printed only on the six-locus report template; for the *IRS1* and *CYP2C9* terms of the SU efficacy rule it means that the term was absent from the values four-locus reports issued, although the locus was assayed. Rows marked “reconstructed” are not in the appendix, which maps *CYP2C9* rs1057910 to SU adverse-reaction risk alone; they were inferred from the values the six-locus reports issued, as reported in the Results. The rosiglitazone efficacy category follows the same *SLCO1B1* rule as the repaglinide efficacy category and is therefore identical to it. The stated basis and evidence level for each row are given in S1 Table. No category has been compared with a measured drug-response outcome in this cohort.

### Derivation of the Interpretation Rulebook

The rulebook was not assembled by us from the published literature, and it was not inferred from the pattern of the issued reports. It was transcribed from a standing appendix that the laboratory attached to every report it issued. That appendix contains the laboratory’s own genotype-to-interpretation table for all assayed loci, with columns for gene and locus, variant class (drug target, metabolism, transport or other), evidence level, the outcome affected (disease risk, efficacy or metabolism), genotype, a reference population frequency, and the interpretation text printed for that genotype. Table 1 reproduces the rows of that table that apply to the loci analysed here, with the wording condensed but no mapping altered. Two qualifications matter for what follows. The copy we were able to recover is the appendix issued with the six-locus panel; the appendix that accompanied four-locus reports was not retrievable, so the four-locus rule set can be established only from the values those reports issued. And two elements of Table 1 are not in the appendix at all and were reconstructed from the issued reports: the mapping of *CYP2C9* rs1057910 to the sulfonylurea efficacy category, which the appendix assigns only to sulfonylurea adverse-reaction risk, and the arithmetic by which contributing loci are combined. Both are identified as reconstructions in Table 1 and in S1 Table.

The appendix grades each pharmacogenetic mapping on the PharmGKB clinical annotation scale, which runs 1A, 1B, 2A, 2B, 3 and 4 in decreasing order of evidential strength [11,17]. Every drug-response mapping used in this study is graded Level 3, which PharmGKB defines as a low level of evidence: a single study, an association that has not been replicated, case reports, non-significant studies, or in vitro or functional evidence. No mapping in Table 1 carries a Level 1 or Level 2 annotation. The disease-risk mappings, that is *TCF7L2*, *IRS1* and the two *CDKAL1* loci to T2DM risk and *IRS1* to insulin resistance risk, carry no PharmGKB level at all; the appendix marks them as resting on locus-specific literature. The rulebook therefore rests on low-level pharmacogenetic evidence for the six drug-response categories and on ungraded literature evidence for the two risk categories. For the loci used here the appendix cites PharmGKB clinical annotations, accessed 24 January 2022, for *TCF7L2* rs7903146, *CYP2C9* *3 and *IRS1* rs1801278 with sulfonylureas, *SLCO1B1* rs4149056 with repaglinide and with rosiglitazone, *CDKAL1* rs7756992 and rs7754840 with DPP-4 inhibitors, and *IRS1* rs1801278 with metformin, together with locus-specific literature for the risk loci. It cites no CPIC or Dutch Pharmacogenetics Working Group recommendation, and none was added when the table was transcribed. The reconstructed *CYP2C9*-to-SU-efficacy mapping carries the evidence level the appendix attaches to *CYP2C9* for sulfonylureas, which is also Level 3.

A row of the appendix was carried into Table 1 when three conditions held: the locus was assayed by at least one of the two panel versions in use; the appendix gave an explicit interpretation for every genotype at that locus for that outcome, so that the mapping was total rather than partial; and the outcome could be expressed on the three-level scale used here. No mapping was added from a source outside the appendix, and no mapping was reworded in a way that changed the category it assigns. Where the evidence behind a mapping is thin, that thinness is recorded in the evidence-level column of Table 1 rather than resolved. No mapping was upgraded, downgraded or dropped on the strength of its evidence, because the object of this study is the rulebook the laboratory actually used rather than a rulebook we would write ourselves.

The appendix uses a five-level result vocabulary: normal, increased, significantly increased, reduced and significantly reduced. Table 1 uses three levels, so “significantly increased” collapses onto “Increased” and “significantly reduced” onto “Reduced.” The collapse was applied to the issued values and to the rule-derived values alike before the two were compared, so it cannot by itself produce disagreement between them.

The appendix prints the genotype-level interpretations for each locus separately and does not state how contributing loci are combined, so the arithmetic for the three composite categories had to be reconstructed. Four precedence rules reproduce the issued values, and they are stated here and in Table 1 so that the rulebook can be applied by others without ambiguity. They are our reconstruction, not a transcription. First, composite T2DM risk takes the highest risk level across the contributing loci, so a record is classified Increased if any contributing locus carries a risk genotype; this is a Boolean OR over those loci. Second, composite DPP-4i efficacy is determined by *CDKAL1* rs7754840, and where rs7754840 is the CG heterozygote, which maps to Normal, *CDKAL1* rs7756992 determines the class; this rule was not stated in the previous version of this manuscript, and reproduces the issued value in 895 of 908 records. Third, for SU efficacy a reduced-function *TCF7L2* or *IRS1* genotype takes precedence over the increase assigned by a reduced-function *CYP2C9* genotype. Fourth, where no rule applied, because the contributing locus had not been assayed or no genotype was returned, the category was left missing rather than imputed. The last column of Table 1 records the panel versions in which each rule was applied to issued reports. For three categories, metformin, repaglinide and rosiglitazone efficacy, a “six-locus only” entry means that the category was printed only on the six-locus report template, not that the rule itself was restricted; for the *IRS1* and *CYP2C9* terms of the SU efficacy rule it means that the term was absent from the values four-locus reports issued, even though the locus was assayed. That difference is a finding of this study rather than a feature stated in the appendix, and it is reported in the Results.

### Statistical analysis

Analyses were performed in Python 3.11.4. Logistic regression models were fitted with statsmodels 0.14.0, and areas under the receiver operating characteristic curve were computed with scikit-learn 1.3.0. Firth penalised likelihood was implemented directly, as iteratively reweighted least squares with the Jeffreys prior correction to the score equation. A two-sided P value below 0.05 was treated as statistically significant.

### Descriptive analysis and quality control

Age was summarized as mean ± standard deviation (SD). Sex, genotypes and interpretive categories were summarized as counts and percentages. Allele and genotype frequencies were obtained by direct counting. Conformity of each locus to HWE was tested with a chi-squared test.

### Concordance between the issued reports and the rulebook

For every record, each of the eight interpretive categories was derived from the recorded genotypes using the rule in Table 1 and compared with the value issued on the report. Laboratory labels on the five-level reporting scale were collapsed onto the three-level scale before comparison. A comparison was made only where both a rule-derived and an issued value existed, so the denominator is 908 records for the five categories issued in every report and 481 for the three issued only in six-locus reports. Concordance is reported for each category as a numerator over a denominator with a percentage, and agreement is quantified by Cohen’s kappa. The SU efficacy category was compared against the rule that each panel version was found to have applied, as described in the Results. Every discordant assignment was then classified by mechanism. For each record carrying a discordance we searched, over the genotypes that record’s panel assayed, for the smallest set of genotype calls whose alteration would make the entire issued interpretation block reproduce exactly under the same rulebook, up to two calls; records for which no such set exists are departures from the rule itself rather than from the genotype recorded beside it.

### Genotype-to-category association analysis

Unadjusted associations between each locus and the category it contributes to were tested with the chi-squared test on the genotype-by-category table. Each category was then dichotomised in the direction the rulebook assigns: “Increased” against the remainder for the categories the rule raises, and “Reduced” against the remainder for SU efficacy and metformin efficacy. Genotype was entered as an explicit additive dose, 0, 1 or 2, of the allele the rulebook treats as the effect allele: T at *TCF7L2* rs7903146, T at *IRS1* rs1801278, G at *CDKAL1* rs7756992, C at *CDKAL1* rs7754840, C (*3) at *CYP2C9* rs1057910 and C at *SLCO1B1* rs4149056. Genotype strings were mapped to that dose explicitly rather than by sort order, so that the coded direction matches the rule. Models were adjusted for age and sex and results are reported as odds ratios (ORs) with 95% confidence intervals (CIs). Several categories are deterministic functions of the genotype entered as the predictor, so the models are quasi-completely separated and unpenalised maximum likelihood does not return finite estimates for all of them. Each model was therefore also fitted by Firth penalised likelihood, with Wald tests and confidence intervals taken from the penalised estimates. Firth estimates are quoted in the text; both sets are given side by side in S4 Table. Gene-gene interactions were examined by adding an interaction term to these models and comparing the fit with a main-effects model by likelihood ratio test, with multiplicity handled by Bonferroni correction as stated in the Results. All models used complete cases, excluding records with missing data on any model variable.

### Polygenic risk score construction and evaluation

A weighted polygenic risk score (PRS) for T2DM was constructed from the four T2DM-associated loci in the rulebook, to test whether the composite T2DM risk category behaves as a cumulative function of the loci that define it. Each locus was assigned a fixed weight, set a priori and not estimated from these data: 1.5 for *TCF7L2* rs7903146, 1.3 for *CDKAL1* rs7756992, 1.25 for *CDKAL1* rs7754840 and 1.2 for *IRS1* rs1801278. The weights order the loci by the relative importance reported for them in the T2DM literature; they are not effect estimates drawn from any single published model, and no source is cited for their exact values because none exists. The score is therefore a weighted count of risk alleles rather than a calibrated risk estimate, and the relative contribution of each locus is fixed by these weights rather than by allele frequency. Genotypes were coded additively as 0, 1 or 2 copies of the risk allele and multiplied by their weights, and the score was analysed on that weighted scale, so odds ratios are per one unit of it.

Three versions of the score were analysed. The primary analysis is restricted to the complete-panel subgroup, that is the records in which all four contributing loci were assayed, and uses complete cases only, so no score in it contains an imputed locus. The sensitivity analysis uses a three-locus score built only from the loci that both panel versions assayed, *TCF7L2* rs7903146, *IRS1* rs1801278 and *CDKAL1* rs7754840, and is applied to the whole cohort; every record then contributes the same loci, so scores are comparable across panel versions. The third version is the whole-cohort four-locus score reported in the previous version of this paper, in which a locus a record’s panel had not assayed contributed zero, which is equivalent to imputing the reference homozygote. It is retained only as the superseded analysis, so that the change is traceable and so that the effect of that imputation can be measured. To measure it, scores were compared between the two panel versions under both the superseded and the sensitivity approach, with a two-sample t test that does not assume equal variances.

Each score was compared with the composite T2DM risk category as issued on the report, by unadjusted logistic regression, and the area under the receiver operating characteristic curve was computed as a consistency index. The issued category is the comparator for all three scores, so that they are compared on one footing, and it is the only informative comparator for the primary analysis. The four-locus score reproduces the rule-derived category exactly, because a record is classified Increased by the rule precisely when at least one of the four contributing loci is non-reference, which is precisely when that score exceeds zero; against the rule-derived category its consistency index is therefore 1.000 by construction and no odds ratio is estimable. The shared-loci score omits *CDKAL1* rs7756992, which the rule still uses, so it does not separate the rule-derived category perfectly: against that category its consistency index is 0.982. The issued and rule-derived categories agree in 902 of the 908 records. Because the score and the category are built from the same loci, this quantity measures the arithmetic agreement between a weighted sum of genotypes and a Boolean rule applied to the same genotypes. It is not a measure of predictive discrimination, it is reported as a consistency index throughout, and no receiver operating characteristic curve is shown. Associations with age (Pearson correlation) and with sex (two-sample t test) were examined for the primary and the sensitivity score. Observed allele frequencies were compared with gnomAD East Asian reference frequencies as a check on ancestry consistency, and both are reported in S2 Table.

## Results

### Cohort characteristics and genetic profile

The final analysis included 908 records for patients with a clinical diagnosis of T2DM, of which 481 came from the six-locus panel and 427 from the four-locus panel. Mean age was 59.1 ± 12.6 years, with a range of 9–93 years, and the cohort was predominantly male (n = 575, 63.3%). Demographic characteristics are shown in Fig 1.

**Fig 1:**
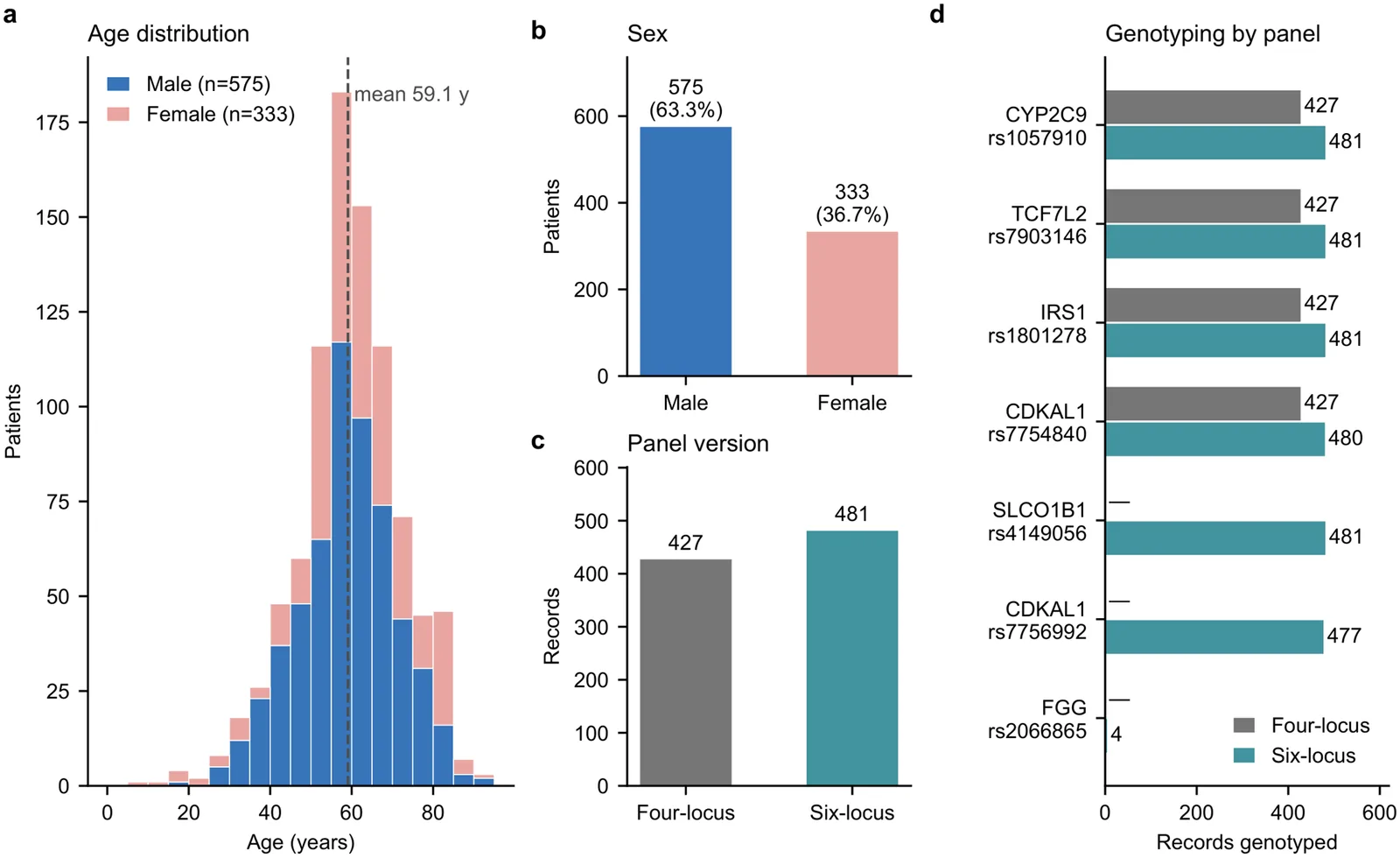
Demographic characteristics of the T2DM study cohort. (a) Age distribution, stacked by sex; the dashed line marks the cohort mean of 59.1 years. Five patients were younger than 18 years. (b) Sex distribution. (c) Number of records issued from each version of the genotyping panel. (d) Number of records genotyped at each locus, by panel version; a dash marks a locus that panel version did not assay. Panels (c) and (d) show that missingness in this cohort is structural, determined by which panel version issued a record rather than by chance. n = 908 records.

Allele frequencies and HWE test results are given in S2 Table. Minor allele frequencies were low for *CYP2C9* rs1057910 (5.0%), *TCF7L2* rs7903146 (4.4%) and *IRS1* rs1801278 (2.1%). Frequencies at the two *CDKAL1* loci were more balanced, at 48.7% for the rs7754840 G allele and 44.1% for the rs7756992 A allele.

Observed frequencies were broadly concordant with gnomAD East Asian reference data (S2 Table). Where they differed, the T2DM risk alleles were the more frequent in this cohort: the *TCF7L2* rs7903146 T allele reached 4.4% against 2.7% in gnomAD East Asians, the *CDKAL1* rs7754840 C allele 51.3% against 38.8%, and the *CDKAL1* rs7756992 G allele 55.9% against 47.4%. Agreement was closer at the locus with no established role in T2DM susceptibility, *SLCO1B1* rs4149056, at 12.4% against 12.7%.

All loci that returned a genotype were tested for HWE (S2 Table). Five of the six analysed loci gave P values above 0.05: rs1057910 P = 0.386, rs7903146 P = 0.548, rs1801278 P = 0.508, rs4149056 P = 0.158 and rs7756992 P = 0.091. *CDKAL1* rs7754840 gave a nominal P of 0.048. That value lies well above the exclusion threshold of 0.05/6 = 0.0083, so the locus was retained; it is also the locus at which the risk allele was most enriched relative to the East Asian reference. Repeating the tests with one record per patient (887 records, of which 886 carried an rs7754840 genotype, 461 an rs4149056 genotype and 457 an rs7756992 genotype) moved the rs7754840 P value to 0.066 and left the other five between 0.076 and 0.549, and allele frequencies changed by less than 0.005 at every locus. *FGG* rs2066865 was excluded from all association analyses because only 4 records carried a genotype at that locus, which is too few for any frequency or equilibrium statistic to carry weight; it was not excluded on quality-control grounds. These characteristics are shown in Fig 2.

**Fig 2:**
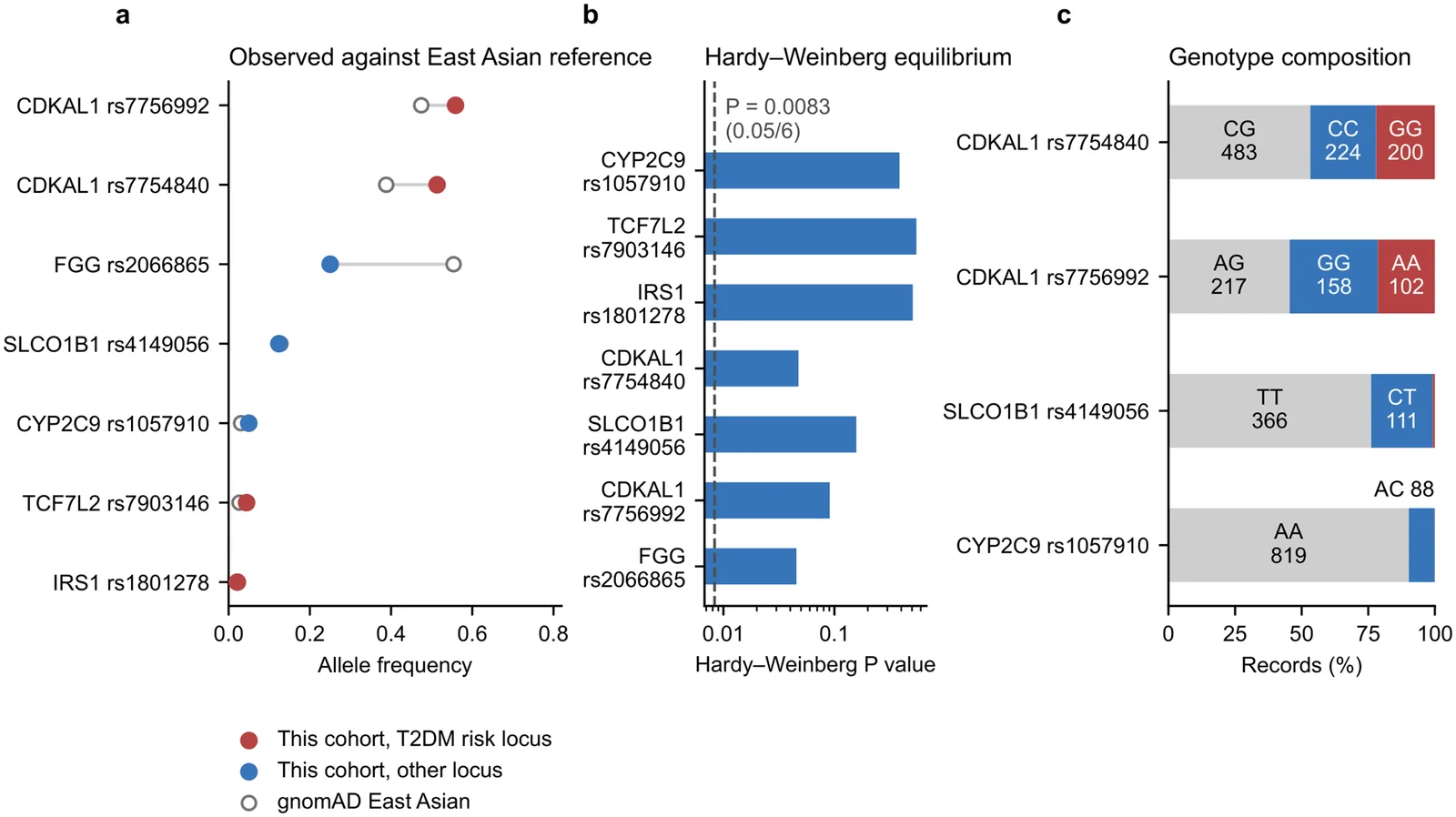
Genetic characteristics of the analysed polymorphisms. (a) Allele frequency observed in this cohort (filled circles) against the gnomAD East Asian reference frequency (open circles) for each locus; red marks the four loci that contribute to the T2DM risk category, at the three risk genes. Observed and reference frequencies agree closely at *SLCO1B1* rs4149056, which has no established role in T2DM susceptibility, whereas the risk alleles are enriched, as expected in a case-only cohort. *FGG* rs2066865 shows the largest gap in the panel, but it was genotyped in only 4 records and no inference is drawn from it. (b) Hardy-Weinberg equilibrium P values from chi-squared tests; the dashed line marks the exclusion threshold of 0.05/6 = 0.0083, and no locus falls below it. (c) Genotype composition of the four loci with balanced or pharmacogenetically relevant frequencies, with the record count of each genotype shown inside its segment, or above the bar where the segment is narrow; the two smallest segments, *CYP2C9* rs1057910 CC (1 record) and *SLCO1B1* rs4149056 CC (4 records), are not labelled.

### Concordance between the issued reports and the Interpretation Rulebook

Agreement between the issued reports and the standardized rulebook was high but not complete. The audit reported here covers all eight interpretive categories. The previous version of this work audited only the four categories that are derived from a single locus and applied one cohort-wide SU rule, and reported 23 of 908 records (2.5%) as discordant; both the denominators and the count change with the wider audit, and the earlier figure is superseded. Five categories were issued in every report and three, metformin, repaglinide and rosiglitazone efficacy, were issued only in six-locus reports, giving 5,983 category assignments across the 908 records. The reason differs between those three: repaglinide and rosiglitazone efficacy depend on *SLCO1B1* rs4149056, which the four-locus panel did not assay, whereas metformin efficacy depends on *IRS1* rs1801278, which it did assay; that category was simply not printed on the four-locus report template. Of these, 5,940 (99.3%) agreed with the rule in Table 1 and 43 (0.7%) did not.

Concordance with the rulebook was 902 of 908 records (99.3%) for T2DM risk, 901 of 908 (99.2%) for insulin resistance risk, 905 of 908 (99.7%) for SU efficacy under the panel-specific rule described below, 905 of 908 (99.7%) for SU ADR risk, 895 of 908 (98.6%) for DPP-4i efficacy, 480 of 481 (99.8%) for metformin efficacy, 476 of 481 (99.0%) for repaglinide efficacy and 476 of 481 (99.0%) for rosiglitazone efficacy (Fig 3a). Cohen’s kappa was 0.90 to 0.99 across the eight categories, and the full agreement statistics, including kappa for each category, are given in S5 Table. At record level, 880 of 908 records (96.9%) agreed with the rulebook on every category and 28 (3.1%) carried at least one discordant assignment. A further 2 records agreed on every category but used a label outside the three-level vocabulary, so 878 of 908 (96.7%) matched the rulebook exactly and 30 (3.3%) departed from it in some way. All records were retained as issued rather than overridden, because the report-issued interpretation is what the treating clinician would have seen. One limit on how these figures should be read follows from how the rulebook was assembled. The per-genotype mappings come from the laboratory’s own appendix, but the rules that combine contributing loci are not in that appendix and were reconstructed to reproduce the issued values, so for the three composite categories, and for the panel-specific sulfonylurea rule, the comparison is in part a measure of how well a reconstructed rule fits the data it was reconstructed from. The five single-locus categories carry no such circularity, and their concordance, from 476 of 481–905 of 908, is of the same order.

**Fig 3:**
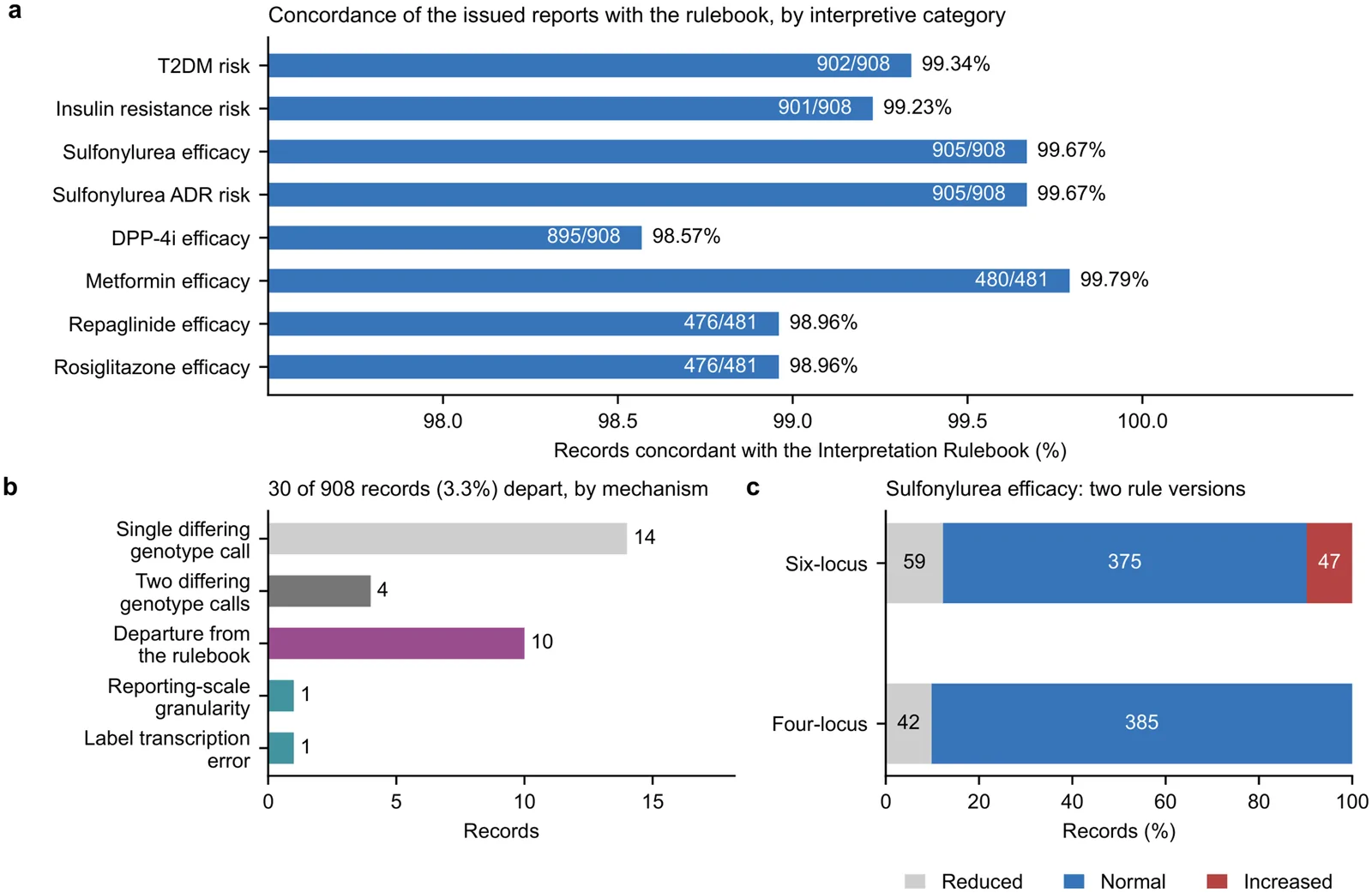
Concordance between the issued reports and the Interpretation Rulebook. (a) Percentage of records whose issued value agreed with the rule in Table 1, for each of the eight interpretive categories, with the numerator and denominator printed inside each bar. The horizontal axis is truncated at 97.5%. Concordance ranged from 895 of 908 (98.6%) for DPP-4i efficacy to 480 of 481 (99.8%) for metformin efficacy, and Cohen’s kappa from 0.90 to 0.99 (S5 Table). Across all 5,983 category assignments, 5,940 (99.3%) matched the rule. (b) The 30 records that departed from the rulebook in any way, classified by mechanism: reproducible by one differing genotype call (14 records), reproducible by two differing calls (4 records), not reproducible by any one- or two-call change (10 records), reporting-scale granularity (1 record) and a transcription error in the issued label (1 record). Every departure is listed with its class in S6 Table. (c) Distribution of the issued SU efficacy value by panel version, with record counts inside the bars. The “Increased” level appears only in reports issued from the six-locus panel, showing that the two panel versions applied different interpretive rules to the same drug; this class of difference is systematic and is handled by panel-specific rules rather than counted as a record error. The forest plot of genotype-to-category associations shown as panel (a) in the previous version has been moved to S1 Fig. n = 908 records.

The 43 discordant assignments and the 2 vocabulary departures were classified by mechanism into six classes, and every departure is listed with its class in S6 Table and summarised in Fig 3b. Class A is a panel-version difference in the interpretive rule; it is systematic rather than record-level, affects SU efficacy alone, and is described in the next paragraph. Class B is reporting-scale granularity and accounted for 1 record, in which a patient with the *CYP2C9* *3/*3 genotype was issued an SU ADR risk of “significantly increased,” a level on the laboratory’s five-level scale that the three-level rulebook collapses to “Increased”; this is not an error. Class C is label transcription and accounted for 1 record, in which T2DM risk was issued as “IncreasedI,” a character appended to “Increased.” Class D accounted for 14 records and 17 discordant assignments in which changing a single locus call reproduces the entire issued interpretation block exactly under the same rulebook, so the interpretation is internally consistent but does not correspond to the genotype recorded alongside it; the loci involved were *CDKAL1* rs7754840 (7 records), *CYP2C9* rs1057910 (2), *IRS1* rs1801278 (2), *CDKAL1* rs7756992 (2) and *SLCO1B1* rs4149056 (1). Class E accounted for 4 records and 11 assignments reproducible by two differing calls; in one of them the issued block corresponds exactly to *CYP2C9* *1/*3 with *SLCO1B1* TT, whereas the genotype fields record *CYP2C9* *1/*1 with *SLCO1B1* CT, so the two results appear transposed. Class F accounted for 10 records and 15 assignments that no one-call or two-call change reproduces, and these are departures from the rulebook itself.

Class F is heterogeneous. In 4 records an *IRS1* CT heterozygote was not carried into insulin resistance risk although the same report assigned SU efficacy as Reduced. In three of the four a *TCF7L2* CT genotype is also present and would assign Reduced SU efficacy on its own, so only one record isolates the *IRS1* term; what all four show is an inconsistency within a single report, the same genotype being read one way for one category and another way for another. In 2 records *CDKAL1* rs7754840 CG was interpreted as GG for DPP-4i efficacy while T2DM risk in the same report followed the CG rule. One record had no rs7754840 result and composite T2DM risk was issued as “Normal” although the assayed rs7756992 AG genotype maps to “Increased,” so an incomplete panel was resolved as if the missing locus were the reference homozygote. One record came from a patient who was tested twice, and its issued DPP-4i efficacy value corresponds to the genotypes of that patient’s other sample rather than to the sample it accompanies. In one further record composite T2DM risk was issued as “Normal” although both assayed *CDKAL1* genotypes, rs7756992 AG and rs7754840 CG, map to “Increased.” In one record six of the eight categories disagreed and no genotype vector reproduced the block. Taken together, classes B and C are reporting artefacts, classes D and E are consistent with a discrepancy between the genotype printed in the report and the genotype used to generate the interpretation, although the audit cannot establish which of the two is correct, and class F is the only group in which an issued interpretation departs from the rules themselves. The heterogeneity quantified here is therefore concentrated in the record-level transfer of genotype into interpretation rather than in disagreement about the rules, with the single exception of the panel-version difference described next.

The SU efficacy category diverged more systematically, and the divergence tracked the genotyping panel. The rules themselves are partly documented: the laboratory appendix from which Table 1 was taken defines SU efficacy on *TCF7L2* and *IRS1*, maps *CYP2C9* rs1057910 to SU ADR risk alone, and defines no “Increased” level for efficacy. Two terms of that documented rule failed to reproduce the issued values in one panel version or the other. First, an “Increased” SU efficacy value was issued in 47 of 481 six-locus records and in 0 of 427 four-locus records, and 46 of those 47 carried a reduced-function *CYP2C9* genotype; the six-locus reports therefore mapped *CYP2C9* *1/*3 and *3/*3 to increased SU efficacy, consistent with the reported improvement in glycaemic response with loss-of-function *CYP2C9* variants [6]. Second, among six-locus records carrying an *IRS1* CT heterozygote with *TCF7L2* CC and a normal-function *CYP2C9* genotype, 20 of 21 were issued “Reduced,” close to the 26 of 27 issued as “Reduced” for the mirror-image genotype *TCF7L2* CT with *IRS1* CC; the same genotype combination was issued “Normal” in all 9 such four-locus records, so the *IRS1* term was absent from the four-locus rule. Applying panel-specific rules, *TCF7L2* alone for four-locus reports and *TCF7L2* with *IRS1* and *CYP2C9* for six-locus reports, reproduced 905 of 908 issued values (99.7%), comprising 427 of 427 four-locus and 478 of 481 six-locus records (99.4%); a single rule applied to every record reproduces at best 869 of 908. Table 1 now records both versions, and the distribution of the issued value by panel version is shown in Fig 3c. The appendix carries no version history, the copy we recovered accompanied six-locus reports, and no report states which version of the algorithm was applied to it. The panel dependence is therefore established by comparing the issued values with the documented rule, not from a dated laboratory amendment, and the *CYP2C9* term of the six-locus rule is a reconstruction from those values rather than a documented mapping.

### Internal consistency of the genotype-to-category mapping

The regression analyses serve one purpose here. They confirm that each term in the rulebook drives the category it was written for, which is a check on the coding rather than a finding about biology. They are reported in full in S4 Table and S1 Fig and only summarised in the text. It is an internal consistency assessment: no independent external, prospective or outcome-based validation was undertaken. In preparing this revision we found an error in the code that produced the estimates reported previously. Genotypes had been coded by sort order of the genotype string, which reversed the dose direction at *CDKAL1* rs7754840 and at *SLCO1B1* rs4149056 and created a spurious fourth genotype level for the single record written “GC.” That is why the previously reported odds ratios at those two loci fell below 1, in the opposite direction to the rule that assigns them. The analysis has been recomputed with an explicit additive dose of the allele the rulebook treats as the effect allele, and with Firth penalised likelihood, which returns finite estimates under separation.

Under the corrected coding every mapping ran in the direction the rulebook assigns and every confidence interval was finite. Firth-penalised odds ratios per additional copy of the effect allele ranged from 15.7 (95% CI 0.93 to 264) for *IRS1* rs1801278 against composite T2DM risk, where that locus is one contributing term among four, to above 10,000 for *CYP2C9* rs1057910 against SU adverse-reaction risk and for *IRS1* rs1801278 against metformin efficacy, where each locus is the only term in the rule. Eleven of the thirteen rows in S4 Table reached P < 0.001; the exceptions are the two composite T2DM risk terms, *TCF7L2* rs7903146 (OR 33.6, 95% CI 2.06 to 548, P = 0.014) and *IRS1* rs1801278 (OR 15.7, 95% CI 0.93 to 264, P = 0.056), for which the composite rule allows another locus to supply the same classification. The magnitudes are not biological effect sizes. Each category is a deterministic function of the genotype entered as the predictor, so the models are quasi-completely separated and each odds ratio records the rigidity of a coding rule. Two rows follow from how the categories were built: repaglinide and rosiglitazone efficacy are assigned by the same *SLCO1B1* rule and were identical in all 481 records assayed at that locus, so they report one finding, and metformin efficacy is estimable once its outcome is dichotomised in the direction the rule assigns, which supersedes the earlier statement that no odds ratio could be obtained for it.

### Polygenic score: internal consistency with the issued T2DM risk category

The primary analysis was restricted to the complete-panel subgroup, the 476 records in which all four contributing loci were assayed, and compares the score with the category as issued. That subgroup is the six-locus panel less five records: four in which *FGG* rs2066865 was assayed in place of *CDKAL1* rs7756992, and one that returned no *CDKAL1* rs7754840 result. One further record could not be coded on the T2DM risk category, leaving 475 records, of which 428 were classified Increased and 47 Normal. Mean score was 2.96 (SD 1.62), with a range of 0 to 7.80, and 9.7% of records scored exactly zero. Each one-unit increase in the score corresponded to a 120-fold increase in the odds of an Increased classification (OR 120, 95% CI 28.9 to 499; P < 0.001), and the consistency index was 0.991. Full results are given in S7 Table and summarized in Fig 4.

**Fig 4:**
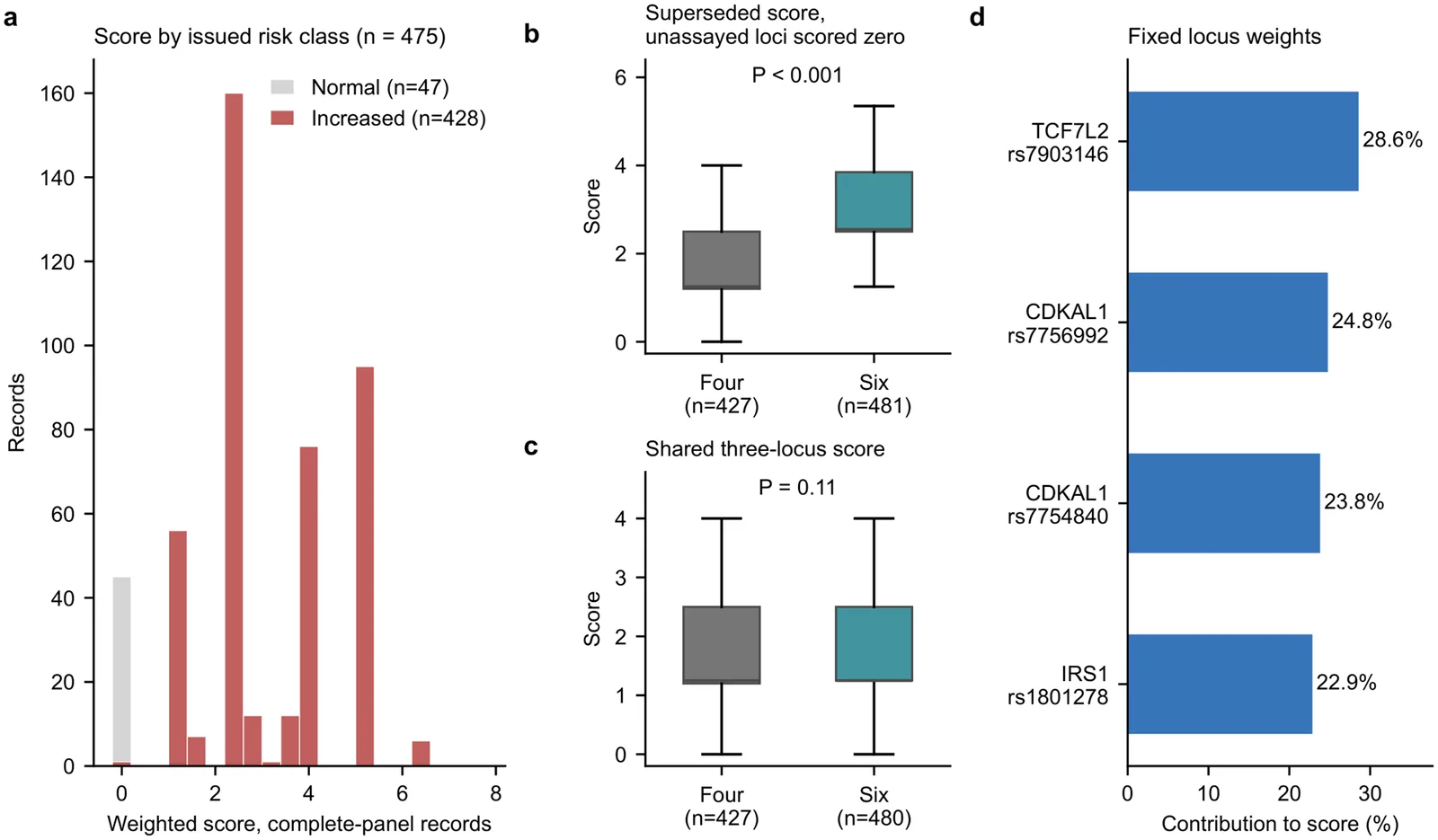
The polygenic score, its dependence on panel version, and its internal consistency with the T2DM risk category as issued. (a) Distribution of the weighted score by issued T2DM risk category in the complete-panel subgroup, which is the primary analysis (n = 475 records). Almost every record classified Normal carries a score of zero, which is why the consistency index is close to one. (b) The superseded whole-cohort score, in which a locus a record’s panel had not assayed contributed zero, plotted by panel version (n = 908 records). Records from the four-locus panel sit lower than records from the six-locus panel (mean 1.41 versus 2.95; t = 17.30, P < 0.001), so part of the spread of that score was created by panel assignment rather than by genotype. (c) The sensitivity score, built only from the three loci that both panel versions assayed, plotted the same way (n = 907 records). The difference between panel versions disappears (1.41 versus 1.52; t = 1.61, P = 0.108). (d) Relative contribution of each locus to the score, fixed by the literature-based weights rather than estimated from these data; *TCF7L2* carries the largest weight, followed by the two *CDKAL1* loci and *IRS1*. Boxes show the median and interquartile range, whiskers 1.5 times the interquartile range. No receiver operating characteristic curve is shown, because the score and the category are built from the same loci; the area under that curve is reported in the text as a consistency index rather than as discrimination.

The sensitivity analysis used only the three loci that both panel versions assayed. Across the whole cohort this gave 906 analysable records, 754 classified Increased and 152 Normal, a mean score of 1.47 (SD 0.99) with a range of 0 to 5.20, and 19.6% of records scoring exactly zero. The association was of the same order as in the primary analysis (OR 193 per unit, 95% CI 74.1 to 506; P < 0.001), with a consistency index of 0.975. Read together with the superseded analysis, these two scores show how much of the previously reported spread was created by panel assignment. Under the superseded approach, in which an unassayed locus contributed zero, records from the four-locus panel had a mean score of 1.41 (SD 1.04) and records from the six-locus panel 2.95 (SD 1.61) (t = 17.30, P < 0.001). Under the shared-loci score the same comparison gives 1.41 (SD 1.04) against 1.52 (SD 0.93) (t = 1.61, P = 0.108). The difference between panel versions disappears once the score stops depending on which loci a panel assayed, which is direct evidence that part of the earlier distribution reflected the panel a record came from rather than genotype.

The superseded whole-cohort analysis is reported here so that the change is traceable. As published, it gave a mean score of 2.23 (SD 1.58) over 907 records, an odds ratio of 373.28 per unit (95% CI 130.54 to 1067.40; P < 0.001) and a consistency index of 0.990; recomputing it with the single record written “GC” counted as the CG heterozygote, as the corrected coding does, gives an odds ratio of 469 (95% CI 150–1467) and a consistency index of 0.991, which changes nothing in the interpretation. Neither version should be read as a score that is comparable across all 908 records. The sex difference reported previously survives in both new analyses: in the complete-panel subgroup the mean score was 2.82 in records from male patients and 3.18 in records from female patients (P = 0.018), and with the shared-loci score 1.38 against 1.62 (P < 0.001). Neither score was correlated with age (complete-panel r = −0.040, P = 0.38; shared loci r = −0.065, P = 0.052).

### Exploratory gene-gene interaction analysis

Eight gene-gene interaction hypotheses were specified before the interaction models were fitted, and all eight are reported; they are listed individually in S8 Table. Each was tested by adding a multiplicative term to the corresponding main-effects model, adjusted for age and sex, and assessed by likelihood ratio test. Six yielded a test; two did not converge, because only 5 and 23 records respectively carry a non-reference genotype at both loci. Multiplicity was handled by Bonferroni correction over the whole specified family of eight, giving a threshold of 0.05/8 = 0.00625. No interaction meets it. Full results are given in S8 Table and S2 Fig.

The interaction term for the two *CDKAL1* loci, rs7756992 and rs7754840, on composite T2DM risk was sub-multiplicative: interaction OR 0.027 (95% CI 0.0023 to 0.318), likelihood ratio test P = 0.033, in the 476 records assayed at both loci. It does not meet the Bonferroni threshold of 0.05/8 = 0.00625, and although it is the smallest likelihood ratio P of the eight we do not treat it as significant. Its direction is also the opposite of the one reported in the previous version of this manuscript. The synergistic interaction OR of 8.21 reported there arose from the genotype coding error described above, which inverted the dose direction at rs7754840; it is superseded by the estimate given here. No other pair showed an interaction: among the five further tests that converged the smallest likelihood ratio P was 0.33.

A sub-multiplicative term of this kind is what the construction of the category implies, and it is not evidence of biological interaction. Composite T2DM risk is assigned by a Boolean OR over the contributing loci: a record is classified Increased if any contributing locus carries a risk genotype. Two contributing loci are therefore substitutes rather than synergists, because once one of them has moved a record into the Increased class the second cannot move it further. On the logit scale that rule produces an interaction odds ratio below 1 as a matter of arithmetic. The joint distribution shows the mechanism directly (S9 Table). Every combination of the two *CDKAL1* genotypes is classified Increased except rs7756992 AA with rs7754840 GG, in which the rule classifies 46 of the 54 records as Normal and 45 were issued as Normal. Sparsity compounds the instability of the estimate: the two smallest cells contain 2 records (rs7756992 AA with rs7754840 CC) and 7 records (rs7756992 GG with rs7754840 GG). A Firth-penalised estimate of the same term is 0.032 (95% CI 0.0064 to 0.156, Wald P = 2.2 × 10^−5^), so the direction is not an artefact of the sparse cells; that penalised Wald test is not the test on which significance is judged here, which remains the likelihood ratio test against its Bonferroni threshold. Nor does the stability of the direction make it biological, because the same direction would appear in any dataset to which the rule was applied. We report the term descriptively and draw no biological inference from it.

## Discussion

The contribution of this study is not a new genetic association. It is a description of how genotype is actually turned into an interpretive category of drug response in routine care, and of how consistently that translation holds. The rulebook in Table 1 makes the mapping explicit, and the association analyses confirm only that each rule term drives the category it was written for. Those analyses are internal checks rather than discoveries, and that is why their detail now sits in the supporting information. The same reading applies to the weighted risk score. The score and the category it was compared against are built from the same loci, so the near-perfect consistency index is a statement about arithmetic. It carries no information about how the score would behave against a measured outcome, in an independent sample, or in a population in which the loci were not the ones assayed. What these data can speak to is the reliability of the translation layer itself, and that is where the informative findings lie.

The case for ancestry-matched interpretation rests here on the allele frequencies rather than on the score. Observed frequencies tracked gnomAD East Asian reference data closely at the locus with no established role in T2DM susceptibility, and departed from it in the expected direction at the risk loci. Recent multi-ancestry analyses report that the genetic architecture of T2DM differs across populations, with East Asians showing distinct mechanistic clusters [23,24], and cross-ancestry polygenic scores often transfer poorly [25–27]. The canonical *TCF7L2* rs7903146 risk allele shows the practical consequence. At 4.4% in this cohort, against roughly 30% in European populations [24,28], a rule built around that variant will flag few Chinese patients however well it performs elsewhere. Loci that are common in East Asian populations, such as the two *CDKAL1* variants, carry more of the usable signal in this setting.

The genotype-to-category mappings draw on a maturing pharmacogenomic literature, and it is worth separating what that literature reports from what this study can support. Reduced-function *CYP2C9* alleles have been associated with sulfonylurea-induced hypoglycaemia in meta-analysis [29], and the Dutch Pharmacogenetics Working Group translates evidence of that kind into explicit dosing advice for *CYP2C9* and the sulfonylureas [16]. *SLCO1B1* variants have been associated with raised repaglinide exposure across a wide dose range [30], and with the therapeutic response to thiazolidinediones [31]. A pharmacogenomic cohort study in an East Asian population reports a *CDKAL1*-modulated response to DPP-4 inhibitors [22]. Those are the findings of the cited studies. Any suggestion to start at a lower dose, to prefer an alternative agent, or to monitor more closely belongs to them and to the guidelines built on them; none of it follows from anything measured here. This cohort carries no drug-response outcome at all. Glycaemic response, hypoglycaemia, adverse events, treatment discontinuation and dose modification were not recorded, so no category assigned by the rulebook has been checked against outcome. The gap matters because misclassification is possible in both directions. A “Normal” category is not a guarantee of therapeutic safety, and a “Reduced” category does not establish that a drug will not work. Reading either as a decision in itself could exclude a viable option or create a false sense of security. What the categories offer, at most, is a standardized record of what a genotype report said. Whether they carry clinical information is a question for prospective studies with measured outcomes.

Another consideration is the strength of the evidence the rulebook rests on, which the evidence-level column of Table 1 now makes visible. Every drug-response mapping in it is graded PharmGKB Level 3, the level reserved for a single study, an unreplicated association, or case reports and functional data; none reaches Level 1 or Level 2. The two disease-risk mappings carry no PharmGKB grade at all. A framework built on evidence of that grade can be applied consistently, which is what this study measures, without thereby becoming clinically reliable, which it does not measure. The distinction is worth keeping in view whenever a rule-based layer is proposed for decision support: consistency of application and strength of underlying evidence are separate properties, and only the first is established here.

The two forms of disagreement we found between the reports and the standardized rule are, in our reading, the most transferable result of this study. The 30 of 908 records that departed from the rule in some way is not a data-processing error. It is a measurement of the reporting heterogeneity that motivated the framework, and it indicates the reconciliation burden a prospective implementation would have to absorb. The structured audit locates most of that heterogeneity: in 18 of the 28 records carrying a genuine discordance the issued interpretation is internally consistent with the rulebook but corresponds to a genotype vector differing from the one printed beside it, which points at the transfer of a result between fields rather than at the interpretive rules. Genotype agreement between repeat records of the same patient was 91 of 94 locus-level comparisons, so the assay itself is not perfectly reproducible either, and that sets a limit on how much of the residual discordance can be attributed to the interpretation step. The sulfonylurea finding is more pointed. Two panel versions running in the same laboratory over the same period applied different interpretive logic to the same drug, and the difference involves two separate terms of the rule. No version marker in the report would have told a clinician which rule had been used. Interpretation therefore drifts as assays are revised, and a rulebook that is written down and versioned is what makes such drift visible.

The interaction between the two *CDKAL1* loci needs a different reading from the one we offered in the previous version of this manuscript. The corrected analysis reverses its direction, and the reversal is the instructive part. Composite T2DM risk is assigned by a Boolean OR across the contributing loci, so a second risk genotype cannot raise a record that a first has already classified as Increased. A sub-multiplicative interaction on the logit scale is the arithmetic signature of that rule. It is a property of the rulebook, not of *CDKAL1*. The point generalises beyond this pair: an interaction term fitted to a category that a combination rule assigns describes the logic of that rule, and any epistasis it appears to show is an artefact of that logic. Intragenic interaction in complex traits is a live question [32–34], but it cannot be addressed with outcomes of this kind. It would require measured phenotypes, pre-specified interaction tests carried through a stated multiplicity correction, and cohorts large enough to fill the sparse compound-genotype cells; the two smallest here contain 2 and 7 records. We therefore make no claim of intragenic epistasis, and in any case the term does not meet the Bonferroni threshold.

The strengths of this study are its explicit translation framework, its basis in records generated by routine clinical practice rather than by research recruitment, and its focus on an underrepresented population. It is not a population sample: every record exists because a clinician ordered pharmacogenomic testing, and what governed that decision is not recorded, so the cohort should not be read as representative of Chinese patients with type 2 diabetes. However, several limitations must be acknowledged. The most important is that the variables analysed here are genotype-derived interpretive categories, not measured clinical endpoints. No drug-response outcome was recorded for any patient, so our results speak to the internal consistency of the classification system and not to its clinical utility. No independent external, prospective or outcome-based validation was undertaken, and none of the eight categories should be treated as guidance for treatment selection, dosing or monitoring. Additionally, 28 of 908 records (3.1%) carried at least one category that differed from the standardized rule, and 2 further records differed only in the label vocabulary used. We regard this as an informative measure of real-world heterogeneity rather than a processing error. It does mean, however, that the classification layer evaluated here is not a purely deterministic function of genotype. The same caution applies more strongly to the SU efficacy category, for which the two panel versions in use during the study period applied different interpretive rules, so that category is not comparable across the whole cohort.

A further limitation concerns the unit of analysis. Each row in this study is an issued report record, not a person. The 908 records correspond to 887 distinct patients, of whom 20 contributed two or three records each, 41 records in total (4.5%). Repeat records were analysed as issued rather than reconciled, so those patients are counted more than once in the frequency, equilibrium and category-level analyses. Repeating the frequency and equilibrium analyses with a single record per patient left allele frequencies unchanged to within 0.005 and left every HWE P value on the same side of the exclusion threshold, so the effect of this duplication on the results reported here is small. A related limitation is that the assay chemistry, reagent manufacturer and reagent lot were not documented on any report and cannot be recovered, so the genotyping itself cannot be reproduced from the record, however completely the interpretation layer is specified.

Two further limitations follow from the panel structure. Missingness at *SLCO1B1* rs4149056 and *CDKAL1* rs7756992 is determined by which panel version issued a record rather than by chance, and *FGG* rs2066865 was assayed in only four records. The complete-case analyses of those loci therefore describe the later, six-locus subgroup rather than the cohort as a whole. The polygenic score has been restructured in response to this. The primary analysis is now restricted to the 475 records with all four contributing loci assayed, so no score is built from an imputed locus, and a score using only the three loci that both panel versions assayed serves as a sensitivity analysis in which every record contributes the same loci. The whole-cohort score reported previously, in which an unassayed locus contributed zero, is retained only as the superseded analysis. Its distribution differs sharply between the panel versions (t = 17.30, P < 0.001) and that difference disappears under the shared-loci score (t = 1.61, P = 0.108), which shows directly that part of its spread was created by panel assignment rather than by genotype. Two limitations of the score survive this change. It is built from a small set of established SNPs and is not a genome-wide score. And the score and the category it was tested against are built from the same loci, so a consistency index of 0.991 records the arithmetic consistency of the classification rules and carries no information about out-of-sample prediction. Finally, this is a single-centre study of one laboratory’s reports, and the interpretive practice of one laboratory need not generalise to others.

## Conclusions

This study set out to standardize real-world pharmacogenomic data in a Chinese T2DM cohort, and in doing so measured how far such data is from being standard already. The rulebook reproduced 5,940 of the 5,983 category assignments made across the cohort (99.3%), but 28 of 908 records (3.1%) departed from it in at least one category, and a structured audit makes most of those departures consistent with a mismatch between the genotype printed on a report and the genotype its interpretation was generated from. The SU efficacy category was governed by two different rules within a single laboratory between July 2022 and April 2024, with nothing in the report to indicate which applied. The internal consistency statistics reported here, including the near-perfect agreement between the weighted risk score and the risk category as issued, follow from the deterministic construction of the categories. They are not evidence of clinical utility, and no independent external, prospective or outcome-based validation was undertaken. What the study supports is narrower and more practical. Interpretation rules should be written down, versioned, and carried with the result, so that drift is visible to the clinician and to anyone who later pools the data. Prospective studies against measured outcomes remain necessary before genotype-derived categories can guide prescribing.

## Supporting information

S1 Table The Interpretation Rulebook: source, mechanism and evidence level for each genotype-to-category mapping. The mechanism stated in the laboratory report appendix for each genotype-to-category mapping, what each category level classifies, the evidence source, and the PharmGKB evidence level attached to each mapping.
(DOCX)

S2 Table Call rates, genotype and allele frequencies, comparison with gnomAD East Asian reference frequencies, and Hardy-Weinberg equilibrium test results. Per-locus call rates with denominators, genotype and allele counts and frequencies, and HWE chi-squared statistics assessed against the stated exclusion threshold.
(DOCX)

S3 Table Genotyping quality-control summary. Call rates with denominators, indeterminate call counts, repeat-testing concordance, Hardy-Weinberg equilibrium results with and without repeated records, and the quantitative criteria under which a locus would have been excluded.
(DOCX)

S4 Table Genotype-to-category association analyses with explicit allele-dose coding and Firth penalised likelihood. Unadjusted chi-squared statistics, unpenalised maximum-likelihood odds ratios and Firth penalised odds ratios, each with 95% confidence intervals, for every locus against the interpretive category it contributes to, adjusted for age and sex.
(DOCX)

S5 Table Agreement between the issued reports and the Interpretation Rulebook, by interpretive category. For each of the eight interpretive categories, the number of records compared, the number and percentage in agreement with the rule in Table 1, Cohen’s kappa, and the number of discordant assignments.
(DOCX)

S6 Table Records whose issued interpretation departed from the standardized rulebook, with the mechanism of each departure. The 43 discordant category assignments in 28 records, together with 2 further records that departed only in label vocabulary, listed with the relevant genotypes, the value the rule in Table 1 assigns, the value as issued, the mechanism class, and the genotype call or calls that reproduce the issued interpretation block where such a set exists.
(DOCX)

S7 Table Weighted score for the T2DM risk category as issued: primary analysis, sensitivity analysis, and the superseded whole-cohort analysis. The complete-panel primary analysis, the shared-loci sensitivity analysis, the superseded whole-cohort analysis, the dependence of each score on panel version, and associations with age and sex.
(DOCX)

S8 Table Gene-gene interaction analyses, with multiplicity handling. The eight specified gene pairs, with interaction odds ratios and 95% confidence intervals, likelihood ratio test P values, change in the Akaike information criterion, Firth-penalised estimates, the number of records carrying a non-reference genotype at both loci, and the position of each term against the Bonferroni threshold.
(DOCX)

S9 Table Contingency table for *CDKAL1* joint genotypes. Joint distribution of *CDKAL1* rs7756992 and rs7754840 genotypes among the 476 records with complete data for both loci.
(DOCX)

S1 Fig Genotype-to-category associations under the corrected additive coding. Firth-penalised odds ratios with 95% confidence intervals on a logarithmic scale for each locus against the interpretive category it contributes to, adjusted for age and sex. Every mapping runs in the direction the rulebook assigns. The magnitudes reflect the rigidity of a deterministic coding rule and are not biological effect sizes. This forest plot was panel (a) of Fig 3 in the previous version.
(TIF)

S2 Fig Gene-gene interaction terms fitted to the interpretive categories as issued. (a) Firth-penalised interaction odds ratios with 95% confidence intervals on a logarithmic scale for the eight specified gene pairs, from models adjusted for age and sex. No term meets the Bonferroni threshold of 0.05/8 = 0.00625 over the eight specified hypotheses, so none is marked as significant. (b) Change in the Akaike information criterion when the interaction term is added, with the likelihood ratio test P value for each pair; negative values indicate improved fit. (c) Joint distribution of the two *CDKAL1* genotypes among the 476 records assayed at both loci, with counts in each cell. The two cells in bold contain 2 and 7 records. The *CDKAL1* term is sub-multiplicative, which follows arithmetically from assigning composite T2DM risk by a Boolean OR over the contributing loci; it is not evidence of biological interaction.
(TIF)
