# Supplementary material for: Genotype-derived interpretive categories of drug response in 908 pharmacogenomic reports from Chinese patients with type 2 diabetes: A retrospective single-centre study
Source: PLoS One. 2026 Nov 5;21(11):e0351903. doi: 10.1371/journal.pone.0351903 (PMC13739094; doi:10.1371/journal.pone.0351903)
Supplement: Supplementary file 1 [file pone.0351903.s001.docx]

**S1 Table. The Interpretation Rulebook: source, mechanism and evidence level for each genotype-to-category mapping.**

Clinical rationale for each genotype-to-category mapping, the level on the rulebook's three-level scale that each genotype is assigned, and the evidence supporting the mapping.

| **Gene** | **SNP (rsID)** | **Interpretive category** | **Genotype** | **Interpretation as recorded in the report appendix, or reconstructed where noted** | **What the category level denotes** | **Evidence source** | **Evidence level** |
| --- | --- | --- | --- | --- | --- | --- | --- |
| TCF7L2 | rs7903146 | T2DM risk | CC | Normal insulin secretion. | Normal: no contributing risk allele at this locus. | Locus-specific literature [18,19] | Not graded by PharmGKB (disease-susceptibility annotation) |
|  |  |  | CT / TT | Reduced insulin secretion; increased genetic risk. | Increased: at least one risk allele at this locus. | Locus-specific literature [18,19] | Not graded by PharmGKB (disease-susceptibility annotation) |
| TCF7L2 | rs7903146 | Sulfonylurea efficacy | CC | Normal beta-cell response. | Normal: no reduced-response allele at this locus. | PharmGKB clinical annotation, sulfonylureas | PharmGKB level 3 |
|  |  |  | CT / TT | Impaired beta-cell function attenuates the response. | Reduced: at least one reduced-response allele. | PharmGKB clinical annotation, sulfonylureas | PharmGKB level 3 |
| IRS1 | rs1801278 | Insulin resistance risk | CC | Normal insulin sensitivity. | Normal: no contributing risk allele. | Locus-specific literature [20] | Not graded by PharmGKB (disease-susceptibility annotation) |
|  |  |  | CT / TT | Reduced insulin sensitivity; increased genetic risk. | Increased: at least one risk allele. | Locus-specific literature [20] | Not graded by PharmGKB (disease-susceptibility annotation) |
| IRS1 | rs1801278 | T2DM risk | CC | Normal insulin sensitivity. | Normal: no contributing risk allele at this locus. | Locus-specific literature [20] | Not graded by PharmGKB (disease-susceptibility annotation) |
|  |  |  | CT / TT | Reduced insulin sensitivity; increased genetic risk. | Increased: at least one risk allele at this locus. | Locus-specific literature [20] | Not graded by PharmGKB (disease-susceptibility annotation) |
| IRS1 | rs1801278 | Sulfonylurea efficacy; metformin efficacy | CC | Normal insulin sensitivity. | Normal: no reduced-response allele. | PharmGKB clinical annotation, drugs used in diabetes | PharmGKB level 3 |
|  |  |  | CT / TT | Reduced insulin sensitivity attenuates the response. | Reduced: at least one reduced-response allele. | PharmGKB clinical annotation, drugs used in diabetes | PharmGKB level 3 |
| CDKAL1 | rs7756992 | T2DM risk | AA | Normal insulin secretion. | Normal: no contributing risk allele at this locus. | Locus-specific literature | Not graded by PharmGKB (disease-susceptibility annotation) |
|  |  |  | AG / GG | Reduced insulin secretion; increased genetic risk. | Increased: at least one risk allele at this locus. | Locus-specific literature | Not graded by PharmGKB (disease-susceptibility annotation) |
| CDKAL1 | rs7756992 | DPP-4 inhibitor efficacy | AA | Reduced incretin sensitivity (beta-cell defect). | Reduced. | PharmGKB clinical annotation, DPP-4 inhibitors [22] | PharmGKB level 3 |
|  |  |  | AG | Normal incretin sensitivity. | Normal. | PharmGKB clinical annotation, DPP-4 inhibitors [22] | PharmGKB level 3 |
|  |  |  | GG | Increased incretin sensitivity. | Increased. | PharmGKB clinical annotation, DPP-4 inhibitors [22] | PharmGKB level 3 |
| CDKAL1 | rs7754840 | T2DM risk | GG | Normal insulin secretion. | Normal: no contributing risk allele at this locus. | Locus-specific literature | Not graded by PharmGKB (disease-susceptibility annotation) |
|  |  |  | CG / CC | Reduced insulin secretion; increased genetic risk. | Increased: at least one risk allele at this locus. | Locus-specific literature | Not graded by PharmGKB (disease-susceptibility annotation) |
| CDKAL1 | rs7754840 | DPP-4 inhibitor efficacy | GG | Reduced incretin sensitivity. | Reduced. | PharmGKB clinical annotation, DPP-4 inhibitors [21] | PharmGKB level 3 |
|  |  |  | CG | Normal incretin sensitivity. | Normal. | PharmGKB clinical annotation, DPP-4 inhibitors [21] | PharmGKB level 3 |
|  |  |  | CC | Increased incretin sensitivity. | Increased. | PharmGKB clinical annotation, DPP-4 inhibitors [21] | PharmGKB level 3 |
| CYP2C9 | rs1057910 | Sulfonylurea ADR risk | *1/*1 (AA) | Normal metabolism; normal clearance. | Normal. | PharmGKB clinical annotation, sulfonylureas [6] | PharmGKB level 3 |
|  |  |  | *1/*3 (AC) | Intermediate metabolism; slower clearance. | Increased. | PharmGKB clinical annotation, sulfonylureas [6] | PharmGKB level 3 |
|  |  |  | *3/*3 (CC) | Poor metabolism; delayed clearance. | Increased (issued as 'significantly increased' on the laboratory's five-level scale). | PharmGKB clinical annotation, sulfonylureas [6] | PharmGKB level 3 |
| CYP2C9 | rs1057910 | Sulfonylurea efficacy (six-locus reports only; reconstructed) | *1/*1 (AA) | Normal metabolism; standard exposure (reconstructed; not in the appendix). | Normal. | PharmGKB clinical annotation, sulfonylureas [6] | PharmGKB level 3 |
|  |  |  | *1/*3 (AC) / *3/*3 (CC) | Reduced clearance raises exposure. | Increased (reconstructed; not in the appendix); a reduced-response TCF7L2 or IRS1 genotype takes precedence. | PharmGKB clinical annotation, sulfonylureas [6] | PharmGKB level 3 |
| SLCO1B1 | rs4149056 | Repaglinide efficacy; rosiglitazone efficacy | TT | Normal transport function; normal clearance. | Normal. | PharmGKB clinical annotation, repaglinide and rosiglitazone [8] | PharmGKB level 3 |
|  |  |  | CT / CC | Reduced transport; increased drug exposure. | Increased. | PharmGKB clinical annotation, repaglinide and rosiglitazone [8] | PharmGKB level 3 |

*ADR, adverse drug reaction; DPP-4i, dipeptidyl peptidase-4 inhibitor; T2DM, type 2 diabetes mellitus. Reference numbers correspond to the reference list of the main text. The interpretations in column five are those printed in the standing appendix carried by every issued report; the Interpretation Rulebook of Table 1 was taken from that appendix, except for the CYP2C9-to-sulfonylurea-efficacy rows and the rules combining contributing loci, which are not in the appendix and were reconstructed from the values the reports issued. The copy recovered accompanied six-locus reports; the four-locus-era appendix could not be retrieved. Evidence levels are those printed in the same appendix, on the PharmGKB scale (1A, 1B, 2A, 2B, 3, 4, in descending order of support). Every pharmacogenetic mapping used here carries PharmGKB level 3, which denotes a low level of evidence: a single study, an association not replicated, or case reports, non-significant studies, in vitro or functional evidence. The disease-susceptibility mappings carry no PharmGKB level and rest on locus-specific literature. None of these categories has been validated against a measured drug-response outcome, in this cohort or elsewhere in this study.*
