# Supplementary material for: Genotype-derived interpretive categories of drug response in 908 pharmacogenomic reports from Chinese patients with type 2 diabetes: A retrospective single-centre study
Source: PLoS One. 2026 Nov 5;21(11):e0351903. doi: 10.1371/journal.pone.0351903 (PMC13739094; doi:10.1371/journal.pone.0351903)
Supplement: Supplementary file 2 [file pone.0351903.s002.docx]

**S2 Table. Call rates, genotype and allele frequencies, comparison with gnomAD East Asian reference frequencies, and Hardy-Weinberg equilibrium test results.**

| **Gene** | **SNP** | **Call rate** | **Sample size** | **Type** | **Variant** | **Count** | **Frequency (this cohort)** | **gnomAD EAS frequency** | **HWE chi-squared** | **HWE P value** | **HWE status** |
| --- | --- | --- | --- | --- | --- | --- | --- | --- | --- | --- | --- |
| CYP2C9 | rs1057910 | 908/908 (100.0%) | 908 | Genotype | AA | 819 | 0.902 | — | 0.75 | 0.386 | Meets criterion |
| CYP2C9 | rs1057910 | 908/908 (100.0%) | 908 | Genotype | AC | 88 | 0.097 | — | 0.75 | 0.386 | Meets criterion |
| CYP2C9 | rs1057910 | 908/908 (100.0%) | 908 | Genotype | CC | 1 | 0.001 | — | 0.75 | 0.386 | Meets criterion |
| CYP2C9 | rs1057910 | 908/908 (100.0%) | 1816 | Allele | A | 1726 | 0.95 | 0.969 | 0.75 | 0.386 | Meets criterion |
| CYP2C9 | rs1057910 | 908/908 (100.0%) | 1816 | Allele | C | 90 | 0.05 | 0.031 | 0.75 | 0.386 | Meets criterion |
| TCF7L2 | rs7903146 | 908/908 (100.0%) | 908 | Genotype | CC | 829 | 0.913 | — | 0.36 | 0.548 | Meets criterion |
| TCF7L2 | rs7903146 | 908/908 (100.0%) | 908 | Genotype | CT | 78 | 0.086 | — | 0.36 | 0.548 | Meets criterion |
| TCF7L2 | rs7903146 | 908/908 (100.0%) | 908 | Genotype | TT | 1 | 0.001 | — | 0.36 | 0.548 | Meets criterion |
| TCF7L2 | rs7903146 | 908/908 (100.0%) | 1816 | Allele | C | 1736 | 0.956 | 0.973 | 0.36 | 0.548 | Meets criterion |
| TCF7L2 | rs7903146 | 908/908 (100.0%) | 1816 | Allele | T | 80 | 0.044 | 0.027 | 0.36 | 0.548 | Meets criterion |
| IRS1 | rs1801278 | 908/908 (100.0%) | 908 | Genotype | CC | 869 | 0.957 | — | 0.44 | 0.508 | Meets criterion |
| IRS1 | rs1801278 | 908/908 (100.0%) | 908 | Genotype | CT | 39 | 0.043 | — | 0.44 | 0.508 | Meets criterion |
| IRS1 | rs1801278 | 908/908 (100.0%) | 1816 | Allele | C | 1777 | 0.979 | 0.98 | 0.44 | 0.508 | Meets criterion |
| IRS1 | rs1801278 | 908/908 (100.0%) | 1816 | Allele | T | 39 | 0.021 | 0.02 | 0.44 | 0.508 | Meets criterion |
| CDKAL1 | rs7754840 | 907/908 (99.9%) | 907 | Genotype | GG | 200 | 0.221 | — | 3.93 | 0.048 | Meets criterion |
| CDKAL1 | rs7754840 | 907/908 (99.9%) | 907 | Genotype | CG | 483 | 0.533 | — | 3.93 | 0.048 | Meets criterion |
| CDKAL1 | rs7754840 | 907/908 (99.9%) | 907 | Genotype | CC | 224 | 0.247 | — | 3.93 | 0.048 | Meets criterion |
| CDKAL1 | rs7754840 | 907/908 (99.9%) | 1814 | Allele | G | 883 | 0.487 | 0.613 | 3.93 | 0.048 | Meets criterion |
| CDKAL1 | rs7754840 | 907/908 (99.9%) | 1814 | Allele | C | 931 | 0.513 | 0.388 | 3.93 | 0.048 | Meets criterion |
| SLCO1B1 | rs4149056 | 481/481 (100.0%) | 481 | Genotype | TT | 366 | 0.761 | — | 2.0 | 0.158 | Meets criterion |
| SLCO1B1 | rs4149056 | 481/481 (100.0%) | 481 | Genotype | CT | 111 | 0.231 | — | 2.0 | 0.158 | Meets criterion |
| SLCO1B1 | rs4149056 | 481/481 (100.0%) | 481 | Genotype | CC | 4 | 0.008 | — | 2.0 | 0.158 | Meets criterion |
| SLCO1B1 | rs4149056 | 481/481 (100.0%) | 962 | Allele | T | 843 | 0.876 | 0.873 | 2.0 | 0.158 | Meets criterion |
| SLCO1B1 | rs4149056 | 481/481 (100.0%) | 962 | Allele | C | 119 | 0.124 | 0.127 | 2.0 | 0.158 | Meets criterion |
| CDKAL1 | rs7756992 | 477/477 (100.0%) | 477 | Genotype | AA | 102 | 0.214 | — | 2.86 | 0.091 | Meets criterion |
| CDKAL1 | rs7756992 | 477/477 (100.0%) | 477 | Genotype | AG | 217 | 0.455 | — | 2.86 | 0.091 | Meets criterion |
| CDKAL1 | rs7756992 | 477/477 (100.0%) | 477 | Genotype | GG | 158 | 0.331 | — | 2.86 | 0.091 | Meets criterion |
| CDKAL1 | rs7756992 | 477/477 (100.0%) | 954 | Allele | A | 421 | 0.441 | 0.526 | 2.86 | 0.091 | Meets criterion |
| CDKAL1 | rs7756992 | 477/477 (100.0%) | 954 | Allele | G | 533 | 0.559 | 0.474 | 2.86 | 0.091 | Meets criterion |
| FGG | rs2066865 | 4/4 (100.0%) | 4 | Genotype | TT | 3 | 0.75 | — | 4.0 | 0.046 | Meets criterion |
| FGG | rs2066865 | 4/4 (100.0%) | 4 | Genotype | CC | 1 | 0.25 | — | 4.0 | 0.046 | Meets criterion |
| FGG | rs2066865 | 4/4 (100.0%) | 8 | Allele | T | 6 | 0.75 | 0.446 | 4.0 | 0.046 | Meets criterion |
| FGG | rs2066865 | 4/4 (100.0%) | 8 | Allele | C | 2 | 0.25 | 0.554 | 4.0 | 0.046 | Meets criterion |

*HWE, Hardy-Weinberg equilibrium; EAS, East Asian. Call rate is the number of records returning a genotype divided by the number of records whose panel version assayed that locus. HWE was assessed with the chi-squared test on 1 degree of freedom; a locus was to be excluded if its call rate fell below 95% or its HWE P value fell below the Bonferroni threshold of 0.05/6 = 0.0083, and no locus met either criterion. CDKAL1 rs7756992 was assayed in 477 of the 481 six-locus records; the other four received an early configuration of that panel which assayed FGG rs2066865 in its place. FGG rs2066865 was excluded from all association analyses because it was genotyped in only 4 records, not on quality-control grounds; it is reported here on the opposite strand to GRCh38 (this assay C/T corresponds to forward-strand G/A) and the reference frequencies have been aligned accordingly. gnomAD East Asian allele frequencies were retrieved from the Ensembl variation endpoint (GRCh38, gnomAD genome population frequencies) on 27 July 2026 and are shown for allele rows only. One rs7754840 record was written 'GC'; G/C and C/G denote the same heterozygous genotype and it is counted here, and in every analysis, within CG.*
