# Supplementary material for: Genotype-derived interpretive categories of drug response in 908 pharmacogenomic reports from Chinese patients with type 2 diabetes: A retrospective single-centre study
Source: PLoS One. 2026 Nov 5;21(11):e0351903. doi: 10.1371/journal.pone.0351903 (PMC13739094; doi:10.1371/journal.pone.0351903)
Supplement: Supplementary file 3 [file pone.0351903.s003.docx]

**S3 Table. Genotyping quality-control summary.**

| **Item** | **Value** |
| --- | --- |
| Genotyping platform | Tianlong TL988 real-time fluorescence PCR system (Xi'an Tianlong Science and Technology, Xi'an, China) |
| Assay method | Real-time fluorescence PCR, performed by Jinan Fuyou Medical Laboratory as part of routine clinical care. The probe chemistry is not stated on any report |
| Assay chemistry, reagent manufacturer, reagent lot | Not recorded on any report; could not be recovered retrospectively |
| Panel versions | Four-locus panel to 28 April 2023; six-locus panel from 29 April 2023. The changeover is recorded in the laboratory line-level export |
| Locus-records attempted | 4,594 (427 four-locus records x 4 loci + 481 six-locus records x 6 loci) |
| Locus-records returning a genotype | 4,593 (99.98%) |
| Per-locus call rate, CYP2C9 rs1057910 | 908/908 (100%) |
| Per-locus call rate, TCF7L2 rs7903146 | 908/908 (100%) |
| Per-locus call rate, IRS1 rs1801278 | 908/908 (100%) |
| Per-locus call rate, CDKAL1 rs7754840 | 907/908 (99.9%) |
| Per-locus call rate, SLCO1B1 rs4149056 | 481/481 six-locus records (100%) |
| Per-locus call rate, CDKAL1 rs7756992 | 477/477 six-locus records in which it was assayed (100%) |
| Indeterminate or failed calls | None recorded in any report |
| Handling of a missing call | Complete-case exclusion from analyses using that locus (one record, CDKAL1 rs7754840) |
| Repeat testing | 20 patients contributed more than one analysed record (19 contributed two, 1 contributed three; 41 records, 4.5% of the cohort) |
| Repeat-record genotype concordance | 91 of 94 pairwise locus comparisons (96.8%) |
| Repeat-record discordances | 3, in 2 patients, all at CDKAL1 loci and all heterozygote versus homozygote |
| Exclusion criterion, low call rate | Call rate below 95% among records whose panel assayed the locus; no locus met this criterion |
| Exclusion criterion, extreme deviation from HWE | Chi-squared P below 0.05/6 = 0.0083; no locus met this criterion |
| Locus excluded for a different reason | FGG rs2066865, genotyped in 4 records only |
| HWE chi-squared P, all 908 records versus one record per patient | 887 patients; per-locus denominators 887, 887, 887, 886, 461 and 457. CYP2C9 rs1057910 0.386/0.400; TCF7L2 rs7903146 0.548/0.549; IRS1 rs1801278 0.508/0.503; CDKAL1 rs7754840 0.048/0.066; SLCO1B1 rs4149056 0.158/0.190; CDKAL1 rs7756992 0.091/0.076. Every locus stayed on the same side of the 0.0083 exclusion threshold and allele frequencies changed by less than 0.005 |

*HWE, Hardy-Weinberg equilibrium. A locus-record is one locus in one report record. Call rate denominators count only the loci that a record's panel version assayed. The exclusion criteria are those stated in the Methods; they were fixed for this analysis rather than registered in advance, and no locus met either. The assay chemistry, reagent manufacturer and reagent lot were not documented on any report and could not be recovered retrospectively, which limits the reproducibility of the genotyping itself and is stated as a limitation in the main text.*
