# Supplementary material for: Genotype-derived interpretive categories of drug response in 908 pharmacogenomic reports from Chinese patients with type 2 diabetes: A retrospective single-centre study
Source: PLoS One. 2026 Nov 5;21(11):e0351903. doi: 10.1371/journal.pone.0351903 (PMC13739094; doi:10.1371/journal.pone.0351903)
Supplement: Supplementary file 4 [file pone.0351903.s004.docx]

**S4 Table. Genotype-to-category association analyses with explicit allele-dose coding and Firth penalised likelihood.**

| **Gene** | **SNP** | **Effect allele** | **Interpretive category** | **Class compared** | **Records** | **Class events** | **Chi-squared** | **Chi-squared P** | **Maximum-likelihood OR (95% CI)** | **Firth-penalised OR (95% CI)** | **Firth P** |
| --- | --- | --- | --- | --- | --- | --- | --- | --- | --- | --- | --- |
| TCF7L2 | rs7903146 | T | T2DM risk | Increased versus other | 908 | 755 | 17.5 | 0.000156 | 9.61e+11 (0 to inf) | 33.6 (2.06 to 548) | 0.0136 |
| TCF7L2 | rs7903146 | T | Sulfonylurea efficacy | Reduced versus other | 908 | 101 | 672.0 | 4.4e-144 | 2915 (382 to 2.22e4) | 1796 (337 to 9566) | 1.63e-18 |
| IRS1 | rs1801278 | T | T2DM risk | Increased versus other | 908 | 755 | 7.05 | 0.00793 | not estimable (separation) | 15.7 (0.934 to 264) | 0.0558 |
| IRS1 | rs1801278 | T | Insulin resistance risk | Increased versus other | 908 | 34 | 716.0 | 9.11e-158 | 6450 (617 to 6.74e4) | 2925 (459 to 1.86e4) | 2.91e-17 |
| IRS1 | rs1801278 | T | Sulfonylurea efficacy | Reduced versus other | 908 | 101 | 152.0 | 1.01e-33 | 26.2 (12.4 to 55.3) | 25.0 (11.9 to 52.4) | 1.7e-17 |
| IRS1 | rs1801278 | T | Metformin efficacy | Reduced versus other | 481 | 27 | 445.0 | 9.87e-99 | not estimable (separation) | 5.55e4 (455 to 6.76e6) | 8.26e-06 |
| CYP2C9 | rs1057910 | C (*3) | Sulfonylurea ADR risk | Increased versus other | 908 | 92 | 875.0 | 9.089999999999999e-191 | 7.25e+14 (0 to inf) | 2.80e4 (1871 to 4.21e5) | 1.23e-13 |
| CDKAL1 | rs7756992 | G | T2DM risk | Increased versus other | 477 | 429 | 176.0 | 5.89e-39 | 92.4 (21.9 to 389) | 71.3 (20.0 to 255) | 4.94e-11 |
| CDKAL1 | rs7756992 | G | DPP-4 inhibitor efficacy | Increased versus other | 477 | 167 | 704.0 | 5.79e-151 | 226 (94.7 to 540) | 193 (83.5 to 445) | 6.6e-35 |
| CDKAL1 | rs7754840 | C | T2DM risk | Increased versus other | 907 | 755 | 613.0 | 6.779999999999998e-134 | 485 (149 to 1584) | 400 (134 to 1190) | 5.37e-27 |
| CDKAL1 | rs7754840 | C | DPP-4 inhibitor efficacy | Increased versus other | 907 | 281 | 1228.0 | 1.06e-264 | 305 (121 to 767) | 268 (111 to 646) | 1.3e-35 |
| SLCO1B1 | rs4149056 | C | Repaglinide efficacy | Increased versus other | 481 | 114 | 454.0 | 2.79e-99 | 1.09e4 (1121 to 1.06e5) | 4651 (786 to 2.75e4) | 1.28e-20 |
| SLCO1B1 | rs4149056 | C | Rosiglitazone efficacy | Increased versus other | 481 | 114 | 454.0 | 2.79e-99 | 1.09e4 (1121 to 1.06e5) | 4651 (786 to 2.75e4) | 1.28e-20 |

*OR, odds ratio, adjusted for age and sex; CI, confidence interval; ADR, adverse drug reaction. Each genotype was coded as the additive dose (0, 1 or 2) of the allele that the Interpretation Rulebook treats as the effect allele, shown in the third column. Because the rulebook makes each category a deterministic function of the genotype, most of these models are quasi-completely separated: the maximum-likelihood estimates are therefore unbounded or unstable and Firth penalised likelihood was used, which returns a finite estimate under separation. These estimates restate the coding rules and are not biological effect sizes. Repaglinide efficacy and rosiglitazone efficacy are derived from SLCO1B1 rs4149056 under the same rule and are identical in all 481 records assayed at that locus; their two rows therefore report one finding. The estimates reported in the previous version of this table used a genotype coding ordered alphabetically by genotype string, which inverted the dose direction at CDKAL1 rs7754840 and SLCO1B1 rs4149056; those estimates are superseded by the values given here.*
