# Supplementary material for: Genotype-derived interpretive categories of drug response in 908 pharmacogenomic reports from Chinese patients with type 2 diabetes: A retrospective single-centre study
Source: PLoS One. 2026 Nov 5;21(11):e0351903. doi: 10.1371/journal.pone.0351903 (PMC13739094; doi:10.1371/journal.pone.0351903)
Supplement: Supplementary file 5 [file pone.0351903.s005.docx]

**S5 Table. Agreement between the issued reports and the Interpretation Rulebook, by interpretive category.**

| **Interpretive category** | **Contributing locus or loci** | **Records compared** | **Concordant** | **Concordance (%)** | **Discordant** | **Cohen kappa** |
| --- | --- | --- | --- | --- | --- | --- |
| T2DM risk | TCF7L2 rs7903146; IRS1 rs1801278; CDKAL1 rs7756992; CDKAL1 rs7754840 | 908 | 902 | 99.34 | 6 | 0.976 |
| Insulin resistance risk | IRS1 rs1801278 | 908 | 901 | 99.23 | 7 | 0.900 |
| Sulfonylurea efficacy | TCF7L2 rs7903146; IRS1 rs1801278; CYP2C9 rs1057910 | 908 | 905 | 99.67 | 3 | 0.988 |
| Sulfonylurea ADR risk | CYP2C9 rs1057910 | 908 | 905 | 99.67 | 3 | 0.982 |
| DPP-4 inhibitor efficacy | CDKAL1 rs7754840; CDKAL1 rs7756992 | 908 | 895 | 98.57 | 13 | 0.978 |
| Metformin efficacy | IRS1 rs1801278 | 481 | 480 | 99.79 | 1 | 0.981 |
| Repaglinide efficacy | SLCO1B1 rs4149056 | 481 | 476 | 98.96 | 5 | 0.971 |
| Rosiglitazone efficacy | SLCO1B1 rs4149056 | 481 | 476 | 98.96 | 5 | 0.971 |
| All categories | — | 5983 | 5940 | 99.28 | 43 | — |

*Records compared is the number of records in which the category could be both issued and derived; categories that depend on a locus assayed only by the six-locus panel are therefore compared in 481 records. The laboratory's five-level result vocabulary was collapsed onto the rulebook's three-level scale before comparison. Sulfonylurea efficacy was compared against the panel-specific rule described in the main text, which reproduces 427 of 427 four-locus and 478 of 481 six-locus values; a single rule applied to every record reproduces at best 869 of 908. Cohen's kappa is undefined for the pooled row because the label sets differ between categories.*
