# Supplementary material for: Genotype-derived interpretive categories of drug response in 908 pharmacogenomic reports from Chinese patients with type 2 diabetes: A retrospective single-centre study
Source: PLoS One. 2026 Nov 5;21(11):e0351903. doi: 10.1371/journal.pone.0351903 (PMC13739094; doi:10.1371/journal.pone.0351903)
Supplement: Supplementary file 6 [file pone.0351903.s006.docx]

**S6 Table. Records whose issued interpretation departed from the standardized rulebook, with the mechanism of each departure.**

Each row is one interpretive category in one record. Record numbers refer to the row order of the deposited dataset. 'Reconciling genotype change' gives the smallest change to the recorded genotypes that would make the whole issued interpretation block reproduce exactly under the same rulebook.

| **Record** | **Panel** | **Category** | **Rulebook value** | **Value as issued** | **Mechanism** | **Reconciling genotype change** | **Genotypes** |
| --- | --- | --- | --- | --- | --- | --- | --- |
| R0013 | Four-locus | Sulfonylurea ADR risk | Normal | Increased | D. Single differing genotype call | rs1057910 AA→AC | rs7903146 CC; rs1801278 CC; rs7754840 GG; rs1057910 AA |
| R0202 | Four-locus | T2DM risk | Increased | Normal | D. Single differing genotype call | rs7754840 CG→GG | rs7903146 CC; rs1801278 CC; rs7754840 CG; rs1057910 AA |
| R0202 | Four-locus | DPP-4 inhibitor efficacy | Normal | Reduced | D. Single differing genotype call | rs7754840 CG→GG | rs7903146 CC; rs1801278 CC; rs7754840 CG; rs1057910 AA |
| R0222 | Four-locus | Sulfonylurea ADR risk | Increased | Significantly_Increased | B. Reporting-scale granularity | not applicable | rs7903146 CC; rs1801278 CC; rs7754840 CG; rs1057910 CC |
| R0290 | Four-locus | T2DM risk | Normal | Increased | D. Single differing genotype call | rs7754840 GG→CG | rs7903146 CC; rs1801278 CC; rs7754840 GG; rs1057910 AA |
| R0290 | Four-locus | DPP-4 inhibitor efficacy | Reduced | Normal | D. Single differing genotype call | rs7754840 GG→CG | rs7903146 CC; rs1801278 CC; rs7754840 GG; rs1057910 AA |
| R0314 | Four-locus | DPP-4 inhibitor efficacy | Normal | Reduced | F. Departure from the rulebook | none within two calls | rs7903146 CC; rs1801278 CC; rs7754840 CG; rs1057910 AA |
| R0317 | Four-locus | DPP-4 inhibitor efficacy | Normal | Reduced | F. Departure from the rulebook | none within two calls | rs7903146 CC; rs1801278 CC; rs7754840 CG; rs1057910 AA |
| R0332 | Four-locus | DPP-4 inhibitor efficacy | Increased | Normal | D. Single differing genotype call | rs7754840 CC→CG | rs7903146 CC; rs1801278 CC; rs7754840 CC; rs1057910 AA |
| R0333 | Four-locus | Insulin resistance risk | Increased | Normal | D. Single differing genotype call | rs1801278 CT→CC | rs7903146 CC; rs1801278 CT; rs7754840 CC; rs1057910 AA |
| R0334 | Four-locus | Insulin resistance risk | Normal | Increased | D. Single differing genotype call | rs1801278 CC→CT | rs7903146 CC; rs1801278 CC; rs7754840 CC; rs1057910 AA |
| R0341 | Four-locus | Sulfonylurea ADR risk | Normal | Increased | D. Single differing genotype call | rs1057910 AA→AC | rs7903146 CC; rs1801278 CC; rs7754840 CG; rs1057910 AA |
| R0463 | Six-locus | Insulin resistance risk | Increased | Normal | F. Departure from the rulebook | none within two calls | rs7903146 CT; rs1801278 CT; rs7756992 GG; rs7754840 CG; rs1057910 AA; rs4149056 TT |
| R0480 | Six-locus | Insulin resistance risk | Increased | Normal | F. Departure from the rulebook | none within two calls | rs7903146 CC; rs1801278 CT; rs7756992 AG; rs7754840 CG; rs1057910 AA; rs4149056 TT |
| R0491 | Six-locus | T2DM risk | Increased | Normal | D. Single differing genotype call | rs7754840 CG→GG | rs7903146 CC; rs1801278 CC; rs7756992 AA; rs7754840 CG; rs1057910 AA; rs4149056 CT |
| R0507 | Six-locus | Sulfonylurea efficacy | Normal | Increased | E. Two differing genotype calls | rs1057910 AA→AC, rs4149056 CT→TT | rs7903146 CC; rs1801278 CC; rs7756992 AG; rs7754840 GG; rs1057910 AA; rs4149056 CT |
| R0507 | Six-locus | Sulfonylurea ADR risk | Normal | Increased | E. Two differing genotype calls | rs1057910 AA→AC, rs4149056 CT→TT | rs7903146 CC; rs1801278 CC; rs7756992 AG; rs7754840 GG; rs1057910 AA; rs4149056 CT |
| R0507 | Six-locus | Repaglinide efficacy | Increased | Normal | E. Two differing genotype calls | rs1057910 AA→AC, rs4149056 CT→TT | rs7903146 CC; rs1801278 CC; rs7756992 AG; rs7754840 GG; rs1057910 AA; rs4149056 CT |
| R0507 | Six-locus | Rosiglitazone efficacy | Increased | Normal | E. Two differing genotype calls | rs1057910 AA→AC, rs4149056 CT→TT | rs7903146 CC; rs1801278 CC; rs7756992 AG; rs7754840 GG; rs1057910 AA; rs4149056 CT |
| R0508 | Six-locus | T2DM risk | Increased | Normal | F. Departure from the rulebook | none within two calls | rs7903146 CC; rs1801278 CC; rs7756992 AG; rs1057910 AA; rs4149056 TT |
| R0538 | Six-locus | DPP-4 inhibitor efficacy | Increased | Normal | D. Single differing genotype call | rs7754840 CC→CG | rs7903146 CT; rs1801278 CC; rs7756992 AG; rs7754840 CC; rs1057910 AA; rs4149056 CT |
| R0556 | Six-locus | Insulin resistance risk | Increased | Normal | F. Departure from the rulebook | none within two calls | rs7903146 CT; rs1801278 CT; rs7756992 GG; rs7754840 CC; rs1057910 AA; rs4149056 TT |
| R0558 | Six-locus | DPP-4 inhibitor efficacy | Reduced | Normal | F. Departure from the rulebook | none within two calls | rs7903146 CC; rs1801278 CC; rs7756992 AA; rs7754840 GG; rs1057910 AA; rs4149056 CT |
| R0567 | Six-locus | Sulfonylurea efficacy | Reduced | Normal | E. Two differing genotype calls | rs7903146 CT→CC, rs7756992 AA→AG | rs7903146 CT; rs1801278 CC; rs7756992 AA; rs7754840 GG; rs1057910 AA; rs4149056 TT |
| R0575 | Six-locus | T2DM risk | Increased | Normal | F. Departure from the rulebook | none within two calls | rs7903146 CC; rs1801278 CC; rs7756992 AG; rs7754840 CG; rs1057910 AC; rs4149056 TT |
| R0633 | Six-locus | Insulin resistance risk | Increased | Normal | F. Departure from the rulebook | none within two calls | rs7903146 CT; rs1801278 CT; rs7756992 GG; rs7754840 CC; rs1057910 AA; rs4149056 CT |
| R0652 | Six-locus | Repaglinide efficacy | Increased | Normal | D. Single differing genotype call | rs4149056 CT→TT | rs7903146 CC; rs1801278 CC; rs7756992 AA; rs7754840 CG; rs1057910 AA; rs4149056 CT |
| R0652 | Six-locus | Rosiglitazone efficacy | Increased | Normal | D. Single differing genotype call | rs4149056 CT→TT | rs7903146 CC; rs1801278 CC; rs7756992 AA; rs7754840 CG; rs1057910 AA; rs4149056 CT |
| R0717 | Six-locus | T2DM risk | Increased | IncreasedI | C. Label transcription error | not applicable | rs7903146 CC; rs1801278 CT; rs7756992 AG; rs7754840 CG; rs1057910 AA; rs4149056 TT |
| R0729 | Six-locus | Repaglinide efficacy | Increased | Normal | E. Two differing genotype calls | rs7756992 AG→AA, rs4149056 CT→TT | rs7903146 CT; rs1801278 CC; rs7756992 AG; rs7754840 CG; rs1057910 AA; rs4149056 CT |
| R0729 | Six-locus | Rosiglitazone efficacy | Increased | Normal | E. Two differing genotype calls | rs7756992 AG→AA, rs4149056 CT→TT | rs7903146 CT; rs1801278 CC; rs7756992 AG; rs7754840 CG; rs1057910 AA; rs4149056 CT |
| R0729 | Six-locus | DPP-4 inhibitor efficacy | Normal | Reduced | E. Two differing genotype calls | rs7756992 AG→AA, rs4149056 CT→TT | rs7903146 CT; rs1801278 CC; rs7756992 AG; rs7754840 CG; rs1057910 AA; rs4149056 CT |
| R0742 | Six-locus | DPP-4 inhibitor efficacy | Increased | Reduced | D. Single differing genotype call | rs7754840 CC→CG | rs7903146 CC; rs1801278 CC; rs7756992 AA; rs7754840 CC; rs1057910 AA; rs4149056 TT |
| R0795 | Six-locus | T2DM risk | Normal | Increased | D. Single differing genotype call | rs7756992 AA→AG | rs7903146 CC; rs1801278 CC; rs7756992 AA; rs7754840 GG; rs1057910 AA; rs4149056 TT |
| R0804 | Six-locus | Insulin resistance risk | Increased | Normal | F. Departure from the rulebook | none within two calls | rs7903146 CC; rs1801278 CT; rs7756992 AG; rs7754840 CC; rs1057910 AA; rs4149056 TT |
| R0804 | Six-locus | Sulfonylurea efficacy | Reduced | Normal | F. Departure from the rulebook | none within two calls | rs7903146 CC; rs1801278 CT; rs7756992 AG; rs7754840 CC; rs1057910 AA; rs4149056 TT |
| R0804 | Six-locus | Repaglinide efficacy | Normal | Increased | F. Departure from the rulebook | none within two calls | rs7903146 CC; rs1801278 CT; rs7756992 AG; rs7754840 CC; rs1057910 AA; rs4149056 TT |
| R0804 | Six-locus | Rosiglitazone efficacy | Normal | Increased | F. Departure from the rulebook | none within two calls | rs7903146 CC; rs1801278 CT; rs7756992 AG; rs7754840 CC; rs1057910 AA; rs4149056 TT |
| R0804 | Six-locus | DPP-4 inhibitor efficacy | Increased | Normal | F. Departure from the rulebook | none within two calls | rs7903146 CC; rs1801278 CT; rs7756992 AG; rs7754840 CC; rs1057910 AA; rs4149056 TT |
| R0804 | Six-locus | Metformin efficacy | Reduced | Normal | F. Departure from the rulebook | none within two calls | rs7903146 CC; rs1801278 CT; rs7756992 AG; rs7754840 CC; rs1057910 AA; rs4149056 TT |
| R0809 | Six-locus | Repaglinide efficacy | Normal | Increased | E. Two differing genotype calls | rs7756992 AG→GG, rs4149056 TT→CT | rs7903146 CC; rs1801278 CC; rs7756992 AG; rs7754840 CG; rs1057910 AA; rs4149056 TT |
| R0809 | Six-locus | Rosiglitazone efficacy | Normal | Increased | E. Two differing genotype calls | rs7756992 AG→GG, rs4149056 TT→CT | rs7903146 CC; rs1801278 CC; rs7756992 AG; rs7754840 CG; rs1057910 AA; rs4149056 TT |
| R0809 | Six-locus | DPP-4 inhibitor efficacy | Normal | Increased | E. Two differing genotype calls | rs7756992 AG→GG, rs4149056 TT→CT | rs7903146 CC; rs1801278 CC; rs7756992 AG; rs7754840 CG; rs1057910 AA; rs4149056 TT |
| R0821 | Six-locus | DPP-4 inhibitor efficacy | Normal | Increased | D. Single differing genotype call | rs7756992 AG→GG | rs7903146 CC; rs1801278 CC; rs7756992 AG; rs7754840 CG; rs1057910 AA; rs4149056 TT |
| R0827 | Six-locus | DPP-4 inhibitor efficacy | Increased | Normal | D. Single differing genotype call | rs7754840 CC→CG | rs7903146 CC; rs1801278 CC; rs7756992 AG; rs7754840 CC; rs1057910 AA; rs4149056 TT |

*Forty-three category assignments in 28 records were discordant with the rulebook, and 2 further records departed only in label vocabulary, giving 30 of 908 records (3.3%) in total. Class B is the laboratory's five-level result scale, on which the CYP2C9 *3/*3 record was issued 'significantly increased' where the three-level rulebook assigns 'increased'. Class C is a typographical error in the issued label. In classes D and E the issued interpretation is internally consistent with the rulebook but corresponds to a genotype vector differing from the one recorded alongside it at one or at two loci. In class F no genotype vector within two calls reproduces the block, so the departure is from the interpretive rule itself. Records were retained as issued rather than overridden, because the issued interpretation is what the treating clinician would have seen.*
