# Supplementary material for: Genotype-derived interpretive categories of drug response in 908 pharmacogenomic reports from Chinese patients with type 2 diabetes: A retrospective single-centre study
Source: PLoS One. 2026 Nov 5;21(11):e0351903. doi: 10.1371/journal.pone.0351903 (PMC13739094; doi:10.1371/journal.pone.0351903)
Supplement: Supplementary file 7 [file pone.0351903.s007.docx]

**S7 Table. Weighted score for the T2DM risk category as issued: primary analysis, sensitivity analysis, and the superseded whole-cohort analysis.**

| **Analysis** | **Score definition** | **Records** | **Increased** | **Normal** | **Mean (SD)** | **Range** | **Score of zero** | **OR per unit (95% CI)** | **P value** | **Consistency index** |
| --- | --- | --- | --- | --- | --- | --- | --- | --- | --- | --- |
| Primary: complete-panel records, all four contributing loci assayed | rs7903146, rs1801278, rs7756992, rs7754840 | 475 | 428 | 47 | 2.96 (1.62) | 0.00 to 7.80 | 9.7% | 120 (28.9 to 499) | 4.3e-11 | 0.991 |
| Sensitivity: loci assayed by both panel versions, whole cohort | rs7903146, rs1801278, rs7754840 | 906 | 754 | 152 | 1.47 (0.99) | 0.00 to 5.20 | 19.6% | 193 (74.1 to 506) | 6.3e-27 | 0.975 |
| Superseded, as reported in the first revision (GC left unmapped) | rs7903146, rs1801278, rs7756992, rs7754840 | 907 | 754 | 153 | 2.23 (1.58) | 0.00 to 7.80 | 16.8% | 373 (130.5 to 1067) | 2.2000000000000004e-28 | 0.99 |
| Superseded, recomputed with the corrected genotype normalisation | rs7903146, rs1801278, rs7756992, rs7754840 | 907 | 754 | 153 | 2.23 (1.58) | 0.00 to 7.80 | 16.6% | 469 (149.9 to 1467) | 4e-26 | 0.991 |

Dependence of the score on panel version

| **Score** | **Panel A** | **n A** | **Mean (SD) A** | **Panel B** | **n B** | **Mean (SD) B** | **t** | **P value** |
| --- | --- | --- | --- | --- | --- | --- | --- | --- |
| Superseded four-locus score, unassayed loci contributing zero | Four-locus | 427 | 1.41 (1.04) | Six-locus | 481 | 2.95 (1.61) | 17.3 | 1.8e-57 |
| Shared three-locus score | Four-locus | 427 | 1.41 (1.04) | Six-locus | 480 | 1.52 (0.93) | 1.61 | 0.108 |

Associations with age and sex

| **Score** | **Records** | **Age, Pearson r** | **P value (age)** | **Mean, male** | **Mean, female** | **P value (sex)** |
| --- | --- | --- | --- | --- | --- | --- |
| Primary, complete-panel records | 476 | -0.04 | 0.381 | 2.82 | 3.18 | 0.018 |
| Sensitivity, loci assayed by both panel versions | 907 | -0.065 | 0.052 | 1.38 | 1.62 | 0.00051 |

*OR, odds ratio; CI, confidence interval. Weights were fixed a priori and not estimated from these data: TCF7L2 rs7903146 1.5, CDKAL1 rs7756992 1.3, CDKAL1 rs7754840 1.25, IRS1 rs1801278 1.2, each multiplied by the additive dose of the risk allele; they order the loci by the relative importance reported for them in the literature and are not effect estimates from any single published model. The outcome in every row is the composite T2DM risk category as issued on the report. Against the rule-derived category the consistency index would be 1.000 by construction, because a record is classified Increased by the rule exactly when the score exceeds zero. The score and the category it is tested against are built from the same loci, so the quantity reported here as a consistency index (the area under the receiver operating characteristic curve) measures the arithmetic consistency of the rulebook and carries no information about out-of-sample prediction. The primary analysis is restricted to records in which all four contributing loci were assayed. The sensitivity analysis uses only the three loci shared by both panel versions and can therefore be computed for the whole cohort. The superseded analysis is the one reported in the previous version of this manuscript, in which unassayed loci contributed zero; the lower panel shows that this made the score depend on panel version, and that the dependence disappears when only shared loci are used. P values for the panel comparison are from Welch's two-sample t test.*
