# Supplementary material for: Genotype-derived interpretive categories of drug response in 908 pharmacogenomic reports from Chinese patients with type 2 diabetes: A retrospective single-centre study
Source: PLoS One. 2026 Nov 5;21(11):e0351903. doi: 10.1371/journal.pone.0351903 (PMC13739094; doi:10.1371/journal.pone.0351903)
Supplement: Supplementary file 8 [file pone.0351903.s008.docx]

**S8 Table. Gene-gene interaction analyses, with multiplicity handling.**

| **Gene pair** | **Interpretive category** | **Pathway** | **Records** | **Interaction OR (95% CI)** | **Likelihood ratio P** | **Change in AIC** | **Firth-penalised interaction OR (95% CI)** | **Firth P** | **Joint carriers** | **Meets Bonferroni threshold (0.05/8 = 0.00625)** |
| --- | --- | --- | --- | --- | --- | --- | --- | --- | --- | --- |
| IRS1 rs1801278 × TCF7L2 rs7903146 | T2DM risk | Insulin signalling and beta-cell function | 908 | did not converge | — | — | 0.00468 (2.90e-05 to 0.757) | 0.0387 | 5 | No |
| TCF7L2 rs7903146 × CDKAL1 rs7754840 | DPP-4 inhibitor efficacy | Beta-cell function and incretin signalling | 907 | 0.265 (0.0251 to 2.8) | 0.326 | 1.04 | 0.176 (0.0247 to 1.26) | 0.0832 | 60 | No |
| CYP2C9 rs1057910 × SLCO1B1 rs4149056 | Sulfonylurea efficacy | Drug metabolism and transport | 481 | 0.161 (0.00326 to 7.93) | 0.37 | 1.20 | 0.142 (0.00715 to 2.8) | 0.199 | 9 | No |
| IRS1 rs1801278 × CDKAL1 rs7756992 | Metformin efficacy | Insulin sensitivity and beta-cell function | 477 | did not converge | — | — | 4.12 (0.153 to 111) | 0.4 | 23 | No |
| CDKAL1 rs7756992 × CDKAL1 rs7754840 | T2DM risk | Intragenic, same gene | 476 | 0.0272 (0.00233 to 0.318) | 0.0332 | -2.53 | 0.0316 (0.00641 to 0.156) | 2.19e-05 | 346 | No |
| TCF7L2 rs7903146 × SLCO1B1 rs4149056 | Repaglinide efficacy | Beta-cell function and drug transport | 481 | unstable (separation) | 0.859 | 1.97 | 0.04 (0.00127 to 1.25) | 0.0671 | 8 | No |
| CYP2C9 rs1057910 × TCF7L2 rs7903146 | Sulfonylurea ADR risk | Drug metabolism and beta-cell function | 908 | unstable (separation) | 1 | 2.00 | 0.102 (0.00105 to 9.88) | 0.328 | 13 | No |
| IRS1 rs1801278 × TCF7L2 rs7903146 | Insulin resistance risk | Insulin signalling and beta-cell function | 908 | unstable (separation) | 0.925 | 1.99 | 0.0184 (5.65e-04 to 0.6) | 0.0246 | 5 | No |

*OR, odds ratio; CI, confidence interval; AIC, Akaike information criterion. The eight hypotheses specified before the interaction models were fitted were IRS1 rs1801278 x TCF7L2 rs7903146 on T2DM risk; TCF7L2 rs7903146 x CDKAL1 rs7754840 on DPP-4 inhibitor efficacy; CYP2C9 rs1057910 x SLCO1B1 rs4149056 on sulfonylurea efficacy; IRS1 rs1801278 x CDKAL1 rs7756992 on metformin efficacy; CDKAL1 rs7756992 x CDKAL1 rs7754840 on T2DM risk; TCF7L2 rs7903146 x SLCO1B1 rs4149056 on repaglinide efficacy; CYP2C9 rs1057910 x TCF7L2 rs7903146 on sulfonylurea adverse-reaction risk; and IRS1 rs1801278 x TCF7L2 rs7903146 on insulin resistance risk. All eight are reported here. Six yield a likelihood ratio test; two do not converge, because only 5 and 23 records respectively carry a non-reference genotype at both loci, which is the quantity given in the Joint carriers column. Models were adjusted for age and sex, and interaction terms were tested by likelihood ratio test against a main-effects model. Multiplicity was handled by Bonferroni correction over the whole specified family of eight, giving a threshold of 0.05/8 = 0.00625; no interaction meets it, the smallest likelihood ratio P being 0.033. A negative change in AIC indicates improved fit when the interaction term is included. The CDKAL1 rs7756992 x rs7754840 term is sub-multiplicative because composite T2DM risk is assigned by a Boolean OR over the contributing loci, so the two loci act as substitutes; an interaction odds ratio below 1 is an arithmetic consequence of that rule rather than evidence of epistasis. The synergistic interaction odds ratio of 8.21 reported in the previous version of this table arose from the genotype coding error described in the note to S4 Table and is superseded. Cell sizes for the CDKAL1 pair are given in S9 Table; the two smallest contain 2 and 7 records.*
