# Supplementary material for: Genotype-derived interpretive categories of drug response in 908 pharmacogenomic reports from Chinese patients with type 2 diabetes: A retrospective single-centre study
Source: PLoS One. 2026 Nov 5;21(11):e0351903. doi: 10.1371/journal.pone.0351903 (PMC13739094; doi:10.1371/journal.pone.0351903)
Supplement: Supplementary file 9 [file pone.0351903.s009.docx]

**S9 Table. Contingency table for CDKAL1 joint genotypes.**

Joint distribution of CDKAL1 rs7756992 and rs7754840 genotypes among the 476 records with complete data for both loci.

|  | **rs7754840 CC** | **rs7754840 CG** | **rs7754840 GG** | **Total** |
| --- | --- | --- | --- | --- |
| rs7756992 AA | 2 (0.4%) | 46 (9.7%) | 54 (11.3%) | 102 (21.4%) |
| rs7756992 AG | 16 (3.4%) | 179 (37.6%) | 21 (4.4%) | 216 (45.4%) |
| rs7756992 GG | 91 (19.1%) | 60 (12.6%) | 7 (1.5%) | 158 (33.2%) |
| Total | 109 (22.9%) | 285 (59.9%) | 82 (17.2%) | 476 (100.0%) |

*Values are n (%). Under the Interpretation Rulebook, rs7756992 AA and rs7754840 GG are the levels that do not contribute to the composite T2DM risk category. Cell sizes below 10 indicate limited power and instability of the interaction estimate reported in S8 Table.*
